# Therapeutic Repair of Sperm Quality Decline Caused by Polytetrafluoroethylene

**DOI:** 10.1002/advs.202505148

**Published:** 2025-07-25

**Authors:** Shiming Gan, Shumin Zhou, Jiaming Zhou, Guanghui Zhang, Jingshou Chen, Rui Liu, Kuan Sun, Sisi Li, Wenjing Xiong, Yujiao Wen, Jianzhong Sheng, Yu Zhang, Jingchao Ren, Youjiang Li, Hefeng Huang, Chen Zhang

**Affiliations:** ^1^ Department of Reproductive Medicine Center for Reproductive Medicine the Fourth Affiliated Hospital of School of Medicine and International School of Medicine International Institutes of Medicine Zhejiang University Yiwu 322000 China; ^2^ Department of Urology & Andrology Sir Run Run Shaw Hospital Zhejiang University School of Medicine Hangzhou 310029 China; ^3^ Institute of Reproduction and Development Shanghai Key Laboratory of Reproduction and Development Obstetrics and Gynecology Hospital Fudan University Shanghai 200032 China; ^4^ Department of Environmental Health College of Preventive Medicine Third Military Medical University (Army Medical University) Chongqing 400038 China; ^5^ Institute of Reproductive Health Tongji Medical College Huazhong University of Science and Technology Wuhan 430030 China; ^6^ Department of Fetal Medicine & Prenatal Diagnosis Center Shanghai Key Laboratory of Maternal Fetal Medicine Shanghai First Maternity and Infant Hospital Obstetrics and Gynecology Hospital of Tongji University Shanghai 201204 China; ^7^ Department of Obstetrics and Gynecology Union Hospital Tongji Medical College Huazhong University of Science and Technology Wuhan 430022 China; ^8^ School of Public Health Chongqing Medical University Chongqing 400016 China; ^9^ Institute of Medical Genetics and Development Key Laboratory of Reproductive Genetics (Ministry of Education) and Women's Hospital Zhejiang University School of Medicine Zhejiang 310058 China; ^10^ Shanghai Key Laboratory of Female Reproductive Endocrine Related Diseases Shanghai 200032 China

**Keywords:** therapeutic repair, microplastics, skap2, sperm quality, spermatogenesis

## Abstract

The alarming prevalence of environmental microplastics has raised global concerns about fertility. However, the detriment of polytetrafluoroethylene (PTFE, Teflon), a widely used microplastic in non‐stick cookware, to sperm quality remains unclear. Here, a high detection rate (46.62%) and bioaccumulation of PTFE in the male urogenital system are reported and the mechanisms of PTFE exposure on male fertility are investigated in both humans and mice and potential therapeutic strategies are explored. These findings reveal that PTFE exposure delays the development of spermatogonia and spermatocytes, disrupts chromosomal synapsis and the DNA damage response, and promotes the apoptosis of spermatocytes. Interestingly, PTFE exposure specifically targets SKAP2 in the haploid spermatid, leading to disruption of the sperm cytoskeleton, abnormal sperm morphology, and decreased sperm motility. Strikingly, therapy targeting SKAP2 remodels sperm cytoskeleton and morphology and restores sperm motility and male fertility in humans and mice. Collectively, these works illustrate the mechanisms of PTFE exposure impairing spermatogenesis and highlight SKAP2 targeting as a promising therapeutic strategy for treating asthenoteratozoospermia in humans.

## Graphical abstract

Exposure to PTFE (teflon), a commonly used microplastic in non‐stick cookware, significantly reduces sperm quality in both humans and mice. Specifically, PTFE induces the disruption of sperm cytoskeleton and F‐actin assembly through targeting SKAP2 in haploid spermatid. While extracellular vesicles‐loading SKAP2 successfully repairs sperm motility and morphology in both humans and mice.

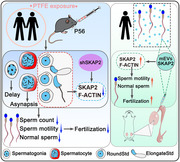

## 1 Introduction

Male infertility is an increasing global concern, affecting individuals and couples worldwide.^[^ ^1^ ^]^ The decline in sperm quality, characterized by decreased sperm count, abnormal morphology, and impaired motility, stems from multiple pathogenic factors, including complex genetic mutations, environmental conditions, and their interactions.^[^ ^2^, ^3^ ^]^ Among these various causes, environmental pollutant has surged in recent years, posing a growing threat to male fertility. Polytetrafluoroethylene (PTFE), a commonly used microplastic that operates a broad temperature range (−180 to 260°C), has extensive applications in medicine and clinical settings, from surgical implants to diagnostic tools.^[^ ^4^, ^5^ ^]^ Notably, PTFE (commonly known as Teflon) is a key component of non‐stick cookware and is frequently used in food processing under high temperatures.^[^ ^6^ ^]^ While its exceptional properties have made PTFE indispensable in modern life, concerns over its long‐term health effects, particularly on male reproductive health, have concurrently intensified. Our epidemiological study suggests a strong correlation between PTFE exposure and reduced sperm quality,^[^ ^7^ ^]^ however, the underlying biological mechanisms are not yet understood.

Milk‐derived extracellular vesicles (mEVs) hold significant potential as biomedical tools, serving both as disease biomarkers and therapeutic agents.^[^ ^8^ ^]^ These vesicles can transport biomolecules, including proteins, lipids, metabolites, and nucleic acids, to target cells.^[^ ^9^ ^]^ Recent studies suggest that environmental pollutants can alter the composition of extracellular vesicles, potentially disrupting the cellular microenvironment and contributing to pathological changes.^[^ ^10^, ^11^ ^]^ In cases of oligoasthenozoospermia, a marked downregulation of extracellular vesicles has been observed.^[^ ^12^ ^]^ Moreover, gene knockout mouse models have demonstrated that the loss of specific extracellular vesicle‐associated proteins can lead to male infertility.^[^ ^13^ ^]^ In vivo and in vitro studies by Murdica et al. revealed that extracellular vesicles isolated from normozoospermic men enhanced sperm motility and promoted rapid sperm activation and capacitation. In contrast, extracellular vesicles derived from asthenozoospermic patients exhibited a negative modulatory effect on sperm quality.^[^ ^14^, ^15^ ^]^ These findings suggest that extracellular vesicle supplementation could serve as a promising therapeutic approach to mitigate the adverse effects of environmental toxins on spermiogenesis. Here, we propose the development of a novel SKAP2‐enriched extracellular vesicle therapy to repair spermiogenesis. SKAP2 (Src kinase‐associated phosphoprotein 2) plays a critical role in orchestrating F‐actin assembly and actin‐mediated asymmetric cytokinesis through its interaction with nucleation‐promoting factors (NPFs) such as WASp family Verprolin‐homologous protein‐2 (WAVE2).^[^ ^16^, ^17^ ^]^ Given that F‐actin assembly is essential for maintaining sperm morphology,^[^ ^18^ ^]^ DNA integrity,^[^ ^19^ ^]^ and motility,^[^ ^20^, ^21^ ^]^ SKAP2‐enriched extracellular vesicles may provide a targeted strategy to counteract spermatogenic defects and enhance male fertility.

In this study, we systematically investigate the impact of PTFE microplastic exposure on sperm quality and fertility in both humans and mouse models. Our findings reveal that PTFE exposure delays the development of spermatogonia and spermatocytes, leading to abnormalities in chromosomal synapsis and an impaired DNA damage response during meiosis. Furthermore, PTFE disrupts the sperm cytoskeleton by down‐regulating SKAP2. Notably, milk‐derived extracellular vesicles containing SKAP2 (mEVs‐SKAP2) enhances sperm motility and DNA integrity in both humans and mice by restoring cytoskeletal integrity. Collectively, our study demonstrates that PTFE exposure compromises sperm function by targeting SKAP2 and highlights extracellular vesicle SKAP2‐based therapy as a promising strategy to improve sperm quality and fertility in cases of PTFE exposure.

## 2 Results

### 2.1 Polytetrafluoroethylene Exposure is Associated with Human Sperm Quality Decline

**Figure 1:**
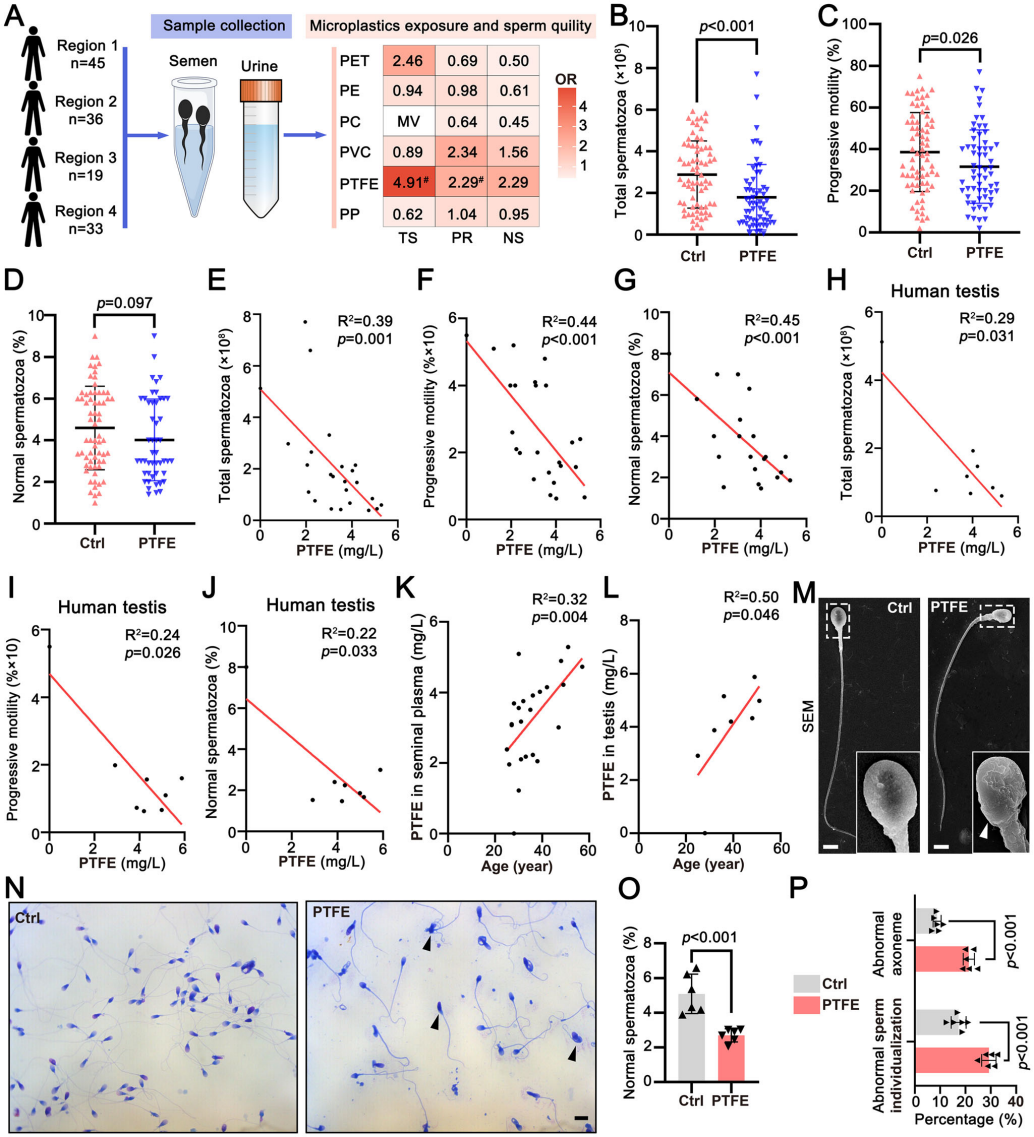
PTFE exposure induces abnormal spermatogenesis in humans. A) A flow diagram outlines the process of volunteer recruitment (left), semen and urine sample collection (middle), and statistical analysis (right). It highlights the association between microplastics exposure and the reduction in semen parameters. Logistic analysis (odds ratio (OR) with 95% confidence interval (CI)) was employed to calculate the OR value for microplastics exposure, adjusting for confounding factors including age, BMI, smoking, alcohol consumption, and exposure sites. Abbreviations, polyethylene terephthalate (PET), polyethylene (PE), polycarbonate (PC), polyvinyl chloride (PVC), polytetrafluoroethylene (PTFE), polypropylene (PP), total spermatozoa (TS), progressive motility (PR), normal spermatozoa (NS). MV denotes missing values, representing cases where statistical analysis was impossible. ^#^ *P* < 0.05. B–D) The quantification of sperm count (B), progressive motility (C), and normal sperm morphology (D) from control and PTFE‐exposed individuals is presented. Data are expressed as mean ± standard deviation (SD). Statistical significance was assessed using a two‐sided Student's *t*‐test. E–G) Linear regression analysis was conducted to examine the relationship between PTFE exposure doses of seminal plasma and their effects on sperm count (E), progressive motility (F), and normal sperm morphology (G). The coefficient of determination (R^2^) is provided, with statistical significance determined using an *F*‐test. H–J) Linear regression analysis was performed to evaluate the association between PTFE exposure doses of testis and their effects on sperm count (H), progressive motility (I), and normal sperm morphology (J). The coefficient of determination (R^2^) is provided, with statistical significance determined using an *F*‐test. K,L) Linear regression analysis evaluated the bioaccumulation of PTFE concentrations in seminal plasma (K), and testis (L) of humans with the increase of age. The coefficient of determination (R^2^) is provided, with statistical significance determined using an *F*‐test. M) Scanning electron microscopy (SEM) images showing the ultrastructure of human epididymal sperm from control and PTFE‐exposed individuals are presented. Insets display higher magnification views of sperm heads, with white arrowheads indicating malformed sperm heads. Scale bar = 5 µm. N) Representative microscopy images of Diff‐Quik‐stained smears of human epididymal sperm from control and PTFE‐exposed individuals are shown. Black arrowheads indicate malformed sperm. Scale bar = 10 µm. O) Histogram depicts the proportion of normal sperm in the human semen from both control and PTFE‐exposed individuals. A total of 500 spermatozoa were counted per individual, and data are presented as mean ± SD. Statistical significance was determined using a two‐sided Student's *t*‐test. P) Quantification of abnormal axonemes (midpiece, principal piece, and endpiece) and sperm individualization from control and PTFE‐exposed individuals. A total of 100 midpieces, 100 principal pieces, and 100 endpieces were counted per individual, with data presented as mean ± SD. Statistical significance was determined using a two‐sided Student's *t*‐test.

To investigate the impact of PTFE microplastic exposure on sperm quality, we collected semen and urine samples from 133 male participants across four different provinces in China and detected the microplastics using Raman microscopy and Pyrolysis‐Gas Chromatography‐Mass Spectrometry (PY‐GC‐MS) (**Figure** **1**A). Notably, we identified PTFE, a widely used hazardous coating material for non‐stick cookware (teflon), was highly associated with semen quality. The participants exposed to PTFE exhibited a severe reduction in total spermatozoa (odds ratio (OR) (95% confidence interval (CI)), 4.91 (0.81, 29.57), *p* = 0.083) and progressive motility (OR (95% CI), 2.29 (0.96, 5.49), *p* = 0.062), with an increased risk of 4.91‐fold and 2.29‐fold, respectively (Figure 1A). Further analysis revealed that PTFE was detected in 46.62% of the human seminal plasma and urine samples (62/133), suggesting that the presence of PTFE in the human body is widespread (Table S1, Supporting Information). The Computer‐assisted sperm analysis (CASA) analysis indicated that the exposure of PTFE leads to reductions of total sperm count (*p* < 0.001), progressive motility (*p* < 0.05), and the morphology of sperm (*p* < 0.10) compared to unexposure of PTFE (Figure 1B–D). To examine the dose‐dependent effects of PTFE exposure on sperm quality, we quantified PTFE concentrations in seminal plasma and testis (Table S2, Supporting Information) using PY‐GC‐MS. Linear regression analysis demonstrated a significant negative correlation between PTFE concentration of seminal plasma and sperm count (*p* < 0.01), progressive motility (*p* < 0.001), and normal sperm proportion (*p* < 0.001) (Figure 1E–G). Further in human testis, we also observed sperm quality decline with the increase of PTFE concentrations (Figure 1H–J). Meanwhile, the bioaccumulation effects of PTFE in seminal plasma and testis of humans intensified with the increase of ages, exacerbating the persistent damage to the male reproductive system (Figure 1K,L). Morphological analysis of sperm samples using Diff‐Quik staining and electron microscopy revealed an increased incidence of acrosomal detachment and head deformity (Figure 1M–O). Additionally, in the middle, principal, and end segments of the flagellum, the proportion of sperm with damaged “9+2” microtubule structures and abnormal sperm individualization significantly increased (Figure 1P). Taken together, these data indicate that exposure to PTFE can contribute to abnormalities in sperm structure, underscoring its potential risk to male reproductive health.

### 2.2 PTFE Penetrates and Disrupts the Blood‐Testis Barrier

To better investigate the mechanism of PTFE's impact on male fertility, we evaluated the structure and properties of PTFE materials. Scanning electron microscopy (SEM) revealed that PTFE particles exhibited irregular shapes, primarily ranging in size from 0 to 2 micrometers (Figure S1A,B, Supporting Information). The ζ‐potentials of the PTFE particles varied from −38.06 to −21.77 mV (Figure S1C, Supporting Information), indicating effective dispersion of the material. Subsequently, eight‐week‐old C57BL/6 male mice were administered varying doses of PTFE through daily intragastric gavage based on the dose detected in human seminal plasma between 0 and 6mg L^−1^ (Figure S1D, Supporting Information). After exposure 30 days, epididymal sperm was collected for analysis. SEM images demonstrated the presence of PTFE in the semen and revealed sperm malformations characterized by deformed heads and bent flagella at doses of 40, 80, and 160 g L^−1^ (Figure S1E, Supporting Information). Furthermore, both SEM (Figure S1F, Supporting Information) and transmission electron microscopy (TEM) (Figure S1G, Supporting Information) indicated that PTFE could penetrate the blood‐testis barrier and accumulate in the spermatogenic tubules, suggesting a direct effect on spermatogenesis. We subsequently quantified the concentration of PTFE in the mouse testis, epididymal tissue, and seminal plasma (Figure S1H–J, Supporting Information). These results showed that the PTFE concentration increased with the gavage dose, further supporting the bioaccumulation of PTFE in male reproductive system.

More importantly, transmission electron microscopy (TEM) analysis revealed that PTFE exposure compromised the integrity of the blood‐testis barrier (Figure S2A, Supporting Information). Disassembly of tight junctions (TJs) between adjacent sertoli cells (SCs) and vacuole formation were observed at PTFE doses of 40 g L^−1^ and higher, while the ultrastructure of the seminiferous epithelium appeared normal, with intact TJs at the control and 20 g L^−1^ groups (Figure S2A, Supporting Information). The disruption of the blood‐testis barrier was further supported by tracking the diffusion of a biotin tracer from beneath the testicular capsules into the seminiferous epithelium (Figure S2B,C, Supporting Information). Additionally, PTFE exposure led to progressive and significant reductions in both body and testis weights in mice at the 160 g L^−1^ dose (Figure S2D,E, Supporting Information). These results suggest that PTFE exposure may adversely affect spermatogenesis by accumulating in the spermatogenic tubules and disrupting the blood‐testis barrier.

### 2.3 PTFE Exposure Affects Spermiogenesis and Sperm Quality

**Figure 2:**
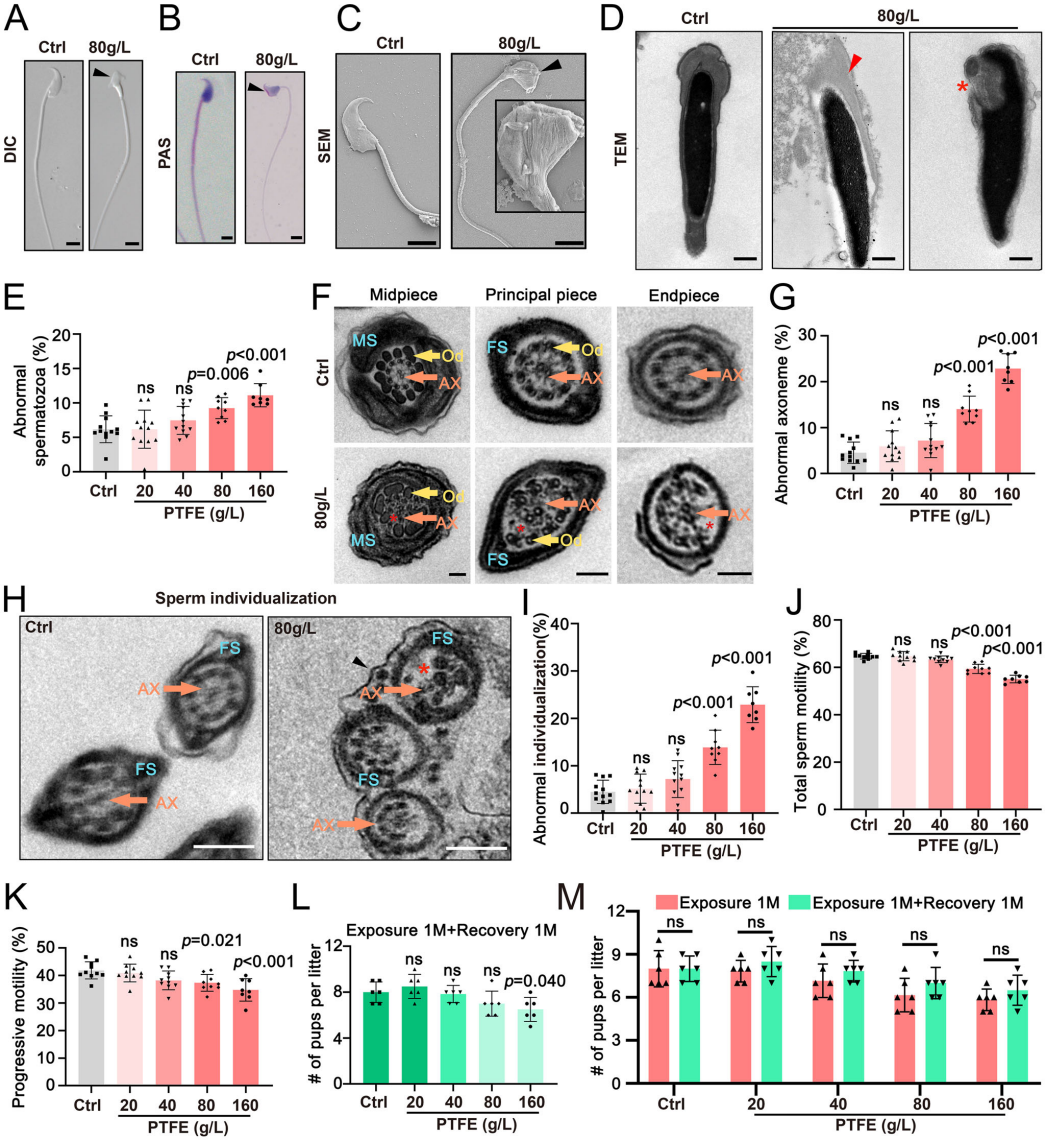
PTFE exposure results in significant spermiogenesis damage in mice. A–C) Representative microscopy images, including differential interference contrast (DIC), H&E staining (HE) staining, and scanning electron microscopy (SEM), of epididymal sperm smears from both control and PTFE‐exposed mice. Black arrowheads denote abnormal sperm heads. Scale bars = 5 µm. D) Transmission electron microscopy (TEM) images illustrate the ultrastructure of sperm heads from control and PTFE‐exposed mice. The red arrowhead highlights an abnormal acrosome and red asterisk indicates a vacuole within the nucleus. Scale bars = 1µm. E) A histogram displaying the percentage of abnormal spermatozoa in control versus PTFE‐exposed mice, with a total of 100 spermatozoa counted for each mouse. Data are presented as mean ± SD. NS = not significant. *p* values were determined using a two‐sided Student's *t*‐test compared with control. F) TEM images depict the ultrastructure of the midpiece, principal piece, and endpiece of sperm flagella from control and PTFE‐exposed mice. Abbreviations include, AX, axoneme, FS, fibrous sheath, Od, outer dense fiber, MS, mitochondrial sheath. The red asterisk indicates absent microtubule doublets in the axoneme. Scale bars = 200 nm. G) Quantification of the percentage of abnormal axonemes (midpiece + principal piece + endpiece) in sperm flagella from control and PTFE‐exposed mice. A total of 100 midpieces, 100 principal pieces, and 100 endpieces were counted per mouse. Data are presented as mean ± SD, with *p* values calculated using a two‐sided Student's *t*‐test compared with control. H) Transmission electron microscopy (TEM) images illustrate sperm individualization in both control and PTFE‐exposed mice. Abbreviations used include AX for axoneme and FS for fiber sheath. The red asterisk denotes the absence of microtubule doublets within the axoneme, and the black arrowhead indicates instance of abnormal sperm individualization. Scale bars represent 200 nm. I) Quantification of the percentage of abnormal sperm individualization in control versus PTFE‐exposed mice was performed, with 100 spermatozoa counted for each mouse. Data are expressed as mean ± SD, and *p* values were determined using a two‐sided Student's *t*‐test compared with control. J,K) Total sperm motility (J) and progressive motility (K) were analysis from control and PTFE‐exposed mice sperm. Data are presented as mean ± SD, with *p* values calculated using a two‐sided Student's *t*‐test compared with control. L) Fertility test results for control and PTFE‐exposed mice following the cessation one month after PTFE exposure one month. Data are expressed as mean ± SD. NS indicates no significant difference. *p*‐values were determined using a two‐sided Student's *t*‐test compared with control. M) Fertility test results for control and PTFE‐exposed mice after one month of exposure and following one‐month recovery period. Data are expressed as mean ± SD. NS indicates no significant difference. *p*‐values were determined using a two‐sided Student's *t*‐test.

Haploid round spermatids undergo a remarkable transformation during spermiogenesis, ultimately developing into streamlined spermatozoa capable of motility.^[^ ^22^ ^]^ In this study, exposure to PTFE at a concentration of 80 g L^−1^ resulted in significant sperm abnormalities, including multiple malformations in epididymal sperm (**Figure** **2**A–C), which were consistent with the teratozoospermia observed in humans. In addition, fertility tests revealed a marked decline in litter size at both 80 and 160 g L^−1^ concentrations (Figure S2F, Supporting Information). Further periodic acid‐Schiff (PAS) staining of testicular sections showed a significant increase in the proportion of abnormal tubules at the 80 and 160 g L^−1^ concentrations (Figure S3A,B, Supporting Information). Additionally, PAS staining of epididymal tissue revealed a gradual decrease in sperm count as PTFE concentration increased (Figure S2C,D, Supporting Information). Collectively, these data suggest that PTFE exposure leads to abnormal spermatogenesis and a decline in sperm quality in mice, which is consistent with results observed in humans.

Transmission electron microscopy (TEM) revealed acrosomal damage and the presence of nuclear vacuoles (Figure 2D). Quantitative analysis demonstrated a marked increase in the proportion of abnormal sperm at PTFE concentrations of 80 and 160 g L^−1^ (Figure 2E). TEM examination of sperm flagella further revealed a missing of ‘9+2’ microtubules in the middle, principal, and end pieces, accompanied by a progressive increase in abnormal axonemes with the increase in PTFE concentrations (Figure 2F,G). Additionally, the proportion of abnormal sperm individualization increasing with PTFE concentrations from 20 to 160 g L^−1^ (Figure 2H,I), indicating that the sperm individualization was hampered. Herein, we employed CASA, and confirmed a gradual decline of both total and progressive sperm motility (Figure 2J,K). Subsequently, we conducted fertility tests after the cessation one month of PTFE exposure (Figure 2L). Strikingly, one month of cessation after exposure to PTFE, the fertility of mice did not significantly recover at concentrations of 80 and 160 g L^−1^ (Figure 2M), suggesting that PTFE‐induced reproductive damage may be persistent, due to the substantial accumulation of PTFE particles in the body and failure of completely eliminated in a short period of time.

**Figure 3:**
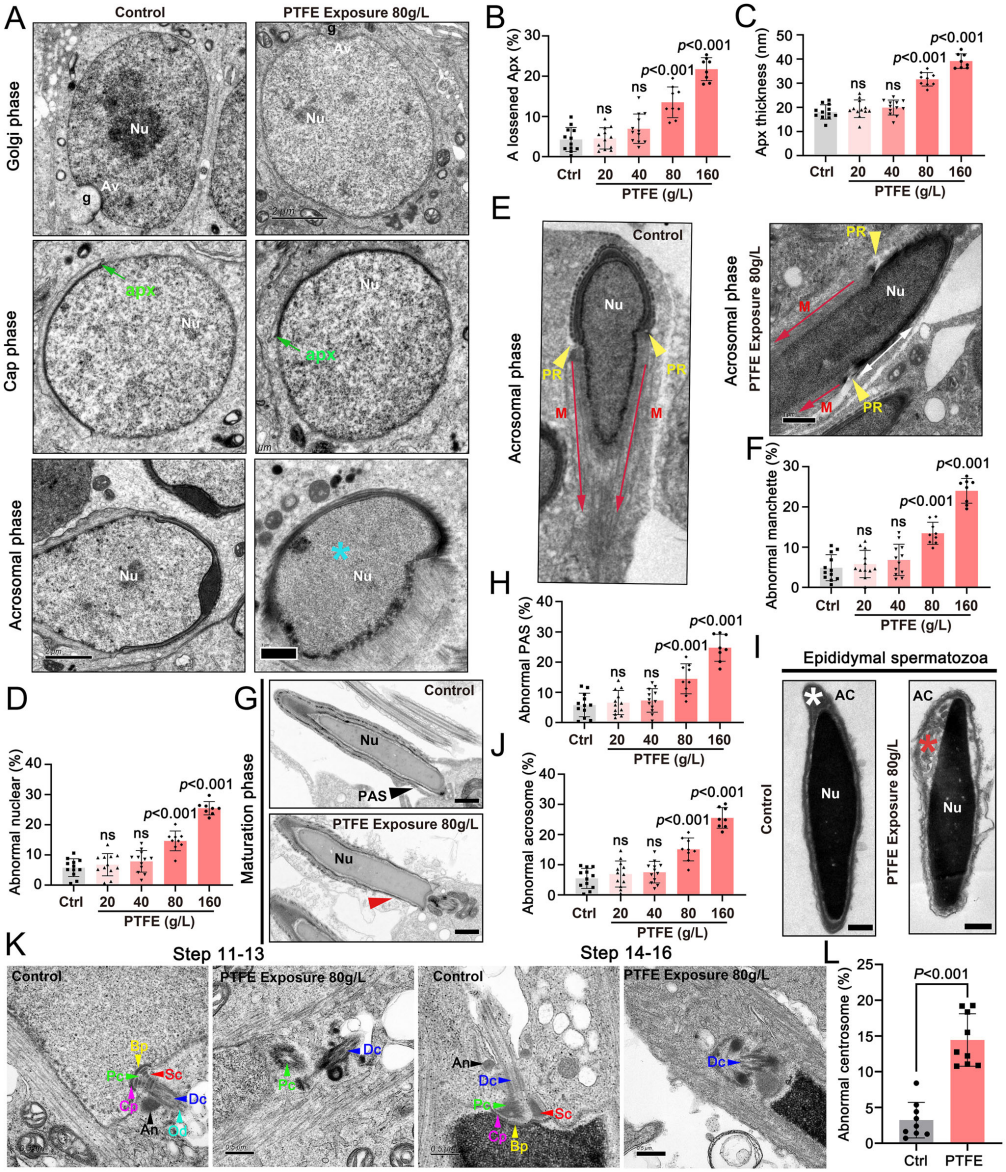
PTFE exposure induces abnormal differentiation of spermatids. A) Transmission electron microscopy (TEM) analysis of spermatid development during the golgi, cap, and acrosomal phases in control and PTFE‐exposed mice. In control spermatids, the acrosome is well‐assembled, whereas it appears disorganized in PTFE‐exposed spermatids. Green arrows indicate the acroplaxome (apx) structure at the cap phase, and the blue asterisk marks abnormal nuclear condensation. Abbreviations, Nu, Nucleus, G, Golgi apparatus, AV, Proacrosomal vesicles, APX, acroplaxome. B–D) Quantification of spermatids exhibiting loosened apx (B), altered apx thickness (C), and abnormal sperm nuclear morphology (D) in control and PTFE‐exposed mice. Five sites of the acroplaxome in each spermatid were randomly selected, and 20 spermatids were counted per mouse. Data are presented as mean ± SD. *p* values were calculated using a two‐sided Student's *t*‐test compared with control. E) TEM analysis of the acrosomal phase in control and PTFE‐exposed mice. Spermatid elongation is mediated by the transient manchette structure. White double‐headed arrows denote the asymmetric manchette structure. Abbreviations, Nu, nucleus, PR, perinuclear ring, M, manchette. F) Percentage of spermatids with abnormal manchette morphology in control and PTFE‐exposed mice, as observed in TEM images (E). A total of 20 spermatids were counted for each mouse. Data are presented as mean ± SD. *p* values were calculated using a two‐sided Student's *t*‐test compared with control. G) TEM analysis at the maturation phase in control and PTFE‐exposed mice. The red arrowhead indicates the absence of the postacrosomal sheath (PAR) structure. Abbreviations, Nu, nucleus, PAR, postacrosomal sheath. H) Percentage of spermatids lacking the PAR structure in control and PTFE‐exposed mice, as observed in TEM images. 20 spermatids were counted for each mouse. Data are presented as mean ± SD. *p* values were calculated using a two‐sided Student's *t*‐test compared with control. I) TEM analysis of epididymal spermatozoa in control and PTFE‐exposed mice. The red asterisk indicates abnormal acrosome structure. Abbreviations, Nu, nucleus, AC, acrosome. J) Percentage of spermatozoa with abnormal acrosome morphology in control and PTFE‐exposed mice. A total of 100 spermatozoa were counted for each mouse. Data are presented as mean ± SD. *p* values were calculated using a two‐sided Student's *t*‐test compared with control. K) TEM analyses of the stepwise development of the centrosome in Control and PTFE exposuree (80g L^−1^) mouse spermatids. The centrosome is well assembled in Control but disorganized in PTFE spermatids. Abbreviations, Bp, basal plate; Cp, capitulum; Sc, segmented column; Pc, proximal centriole; Dc, distal centriole; An, annulus; Od, outer dense fibers. L) Histogram shows the percentage of abnormal centrosomes in Control and PTFE exposuree (80g L^−1^) mouse spermatids. Data are presented as mean ± SD. A total of 200 spermatozoa were counted per mouse. *p* values were calculated using a two‐sided Student's *t*‐test.

To further investigate the effects of PTFE on spermiogenesis, we performed TEM to analyze the structure of ≈20 nm acroplaxome that anchors the acrosome to the nucleus during the cap phase in spermatids. Strikingly, an expanded gap between the inner acrosomal membrane (IAM) and the nuclear envelope (NE) were observed in PTFE‐exposed spermatids (**Figure** **3**A) and the proportion of spermatids exhibiting a loosened acroplaxome structure was significantly higher in PTFE‐exposed spermatids compared to controls (Figure 3B). The thickness of acroplaxome was also significantly increased in PTFE‐exposed spermatids compared to controls (Figure 3C). In addition, there was a gradual increase in the proportion of spermatids with abnormal nuclear morphology during the acrosomal phase (Figure 3D). In the PTFE‐exposed group, although nuclear elongation of round spermatids was in process, abnormal manchette development occurred during nuclear condensation (Figure 3E,F) and a significant loss of the postacrosomal sheath was observed at the maturation stage (Figure 3G,H). In epididymal spermatozoa, a significant increase of abnormal acrosomes at 80 and 160 g L^−1^ groups were also observed (Figure 3I,J). Considering sperm flagella are crucial for maintaining motility, and the centrosome as a key component for the formation of flagella. Thus, we also examined the integrity of the centrosome and observed abnormal organization and missing of the centrosome during spermatid development in PTFE group in comparison with control (Figure 3K,L). Together, these findings suggest that PTFE exposure impairs spermiogenesis, ultimately contributing to the asthenoteratozoospermia.

### 2.4 PTFE Exposure Disrupts Androgen Metabolism in Both Humans and Mice

**Figure 4:**
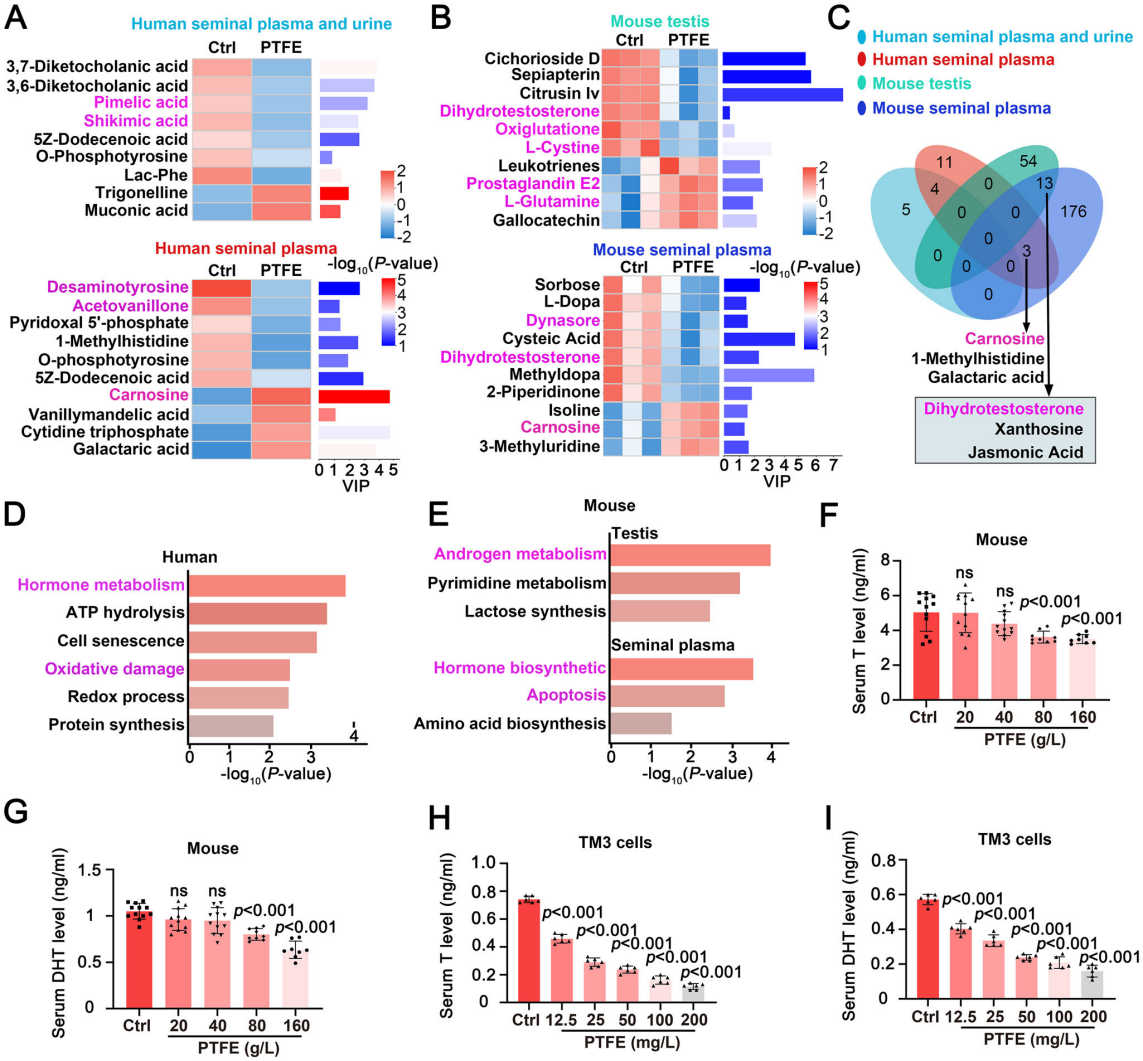
PTFE exposure disrupts androgen metabolism. A) Heat maps illustrate the metabolomic profiles of human seminal plasma and urine. Data from both human seminal plasma and urine samples are presented (controls, 32 subjects, PTFE‐exposed, 37 subjects). The human seminal plasma data include controls (56 subjects) and PTFE‐exposed subjects (17 subjects). The color represents the relative expression of the metabolite, and the value is shown in the gradient color block. VIP, Variable Importance in Projection. The bar length represents the value of the metabolite's contribution to the difference between the two groups. The bar color indicates the significant difference (*P*‐value) of the metabolite between the two groups of samples. B) Heat maps depict the metabolomic profiles of mouse testis and seminal plasma (*n* = 3). The color represents the relative expression of the metabolite, and the value is shown in the gradient color block. VIP, Variable Importance in Projection. The bar length represents the value of the metabolite's contribution to the difference between the two groups. The bar color indicates the significant difference (*P*_value) of the metabolite between the two groups of samples. C) A Venn diagram shows the overlap of metabolites across the four groups from (A,B). D) Gene Ontology (GO) enrichment analysis of differentially expressed metabolites in human seminal plasma. E) GO enrichment analysis of differential metabolites in mouse seminal plasma. F,G) Histograms display serum testosterone (T) and dihydrotestosterone (DHT) levels in control and PTFE‐exposed mice. Testosterone and DHT levels were measured by ELISA, with optical density readings obtained spectrophotometrically at 450 nm (both intra‐cV and inter‐CV < 10%). Data are presented as mean ± SD. NS indicates not significant. *p* values were calculated using Student's *t*‐test (two‐sided) compared with control. H,I) Histograms illustrate serum testosterone (T) and dihydrotestosterone (DHT) levels in TM3 cells. Levels were determined by ELISA, with optical density readings obtained spectrophotometrically at 450 nm (both intra‐cV and inter‐CV < 10%). Data are presented as mean ± SD. NS indicates not significant. *p* values were calculated using Student's *t*‐test (two‐sided) compared with control.

To investigate the impact of PTFE exposure on sperm metabolism, we conducted metabolomic analyses on human seminal plasma and urine. Analysis of differential metabolites between the control group and the PTFE‐exposed group revealed significant dysregulation of metabolites related to amino acid biosynthesis, such as pimelic acid and shikimic acid, as well as metabolites associated with redox reactions, such as deaminotyrosine, acetovanillone, and carnosine (**Figure** **4**A). These findings suggest that PTFE exposure may impair protein synthesis and induce oxidative damage. Subsequently, we analyzed the differential metabolites in the testicular tissue and seminal plasma between the control group and the PTFE exposure group of mice (Figure 4B). The dihydrotestosterone emerging as a notable difference metabolites indicated that PTFE disrupted the androgen metabolism. Venn analysis of the differential metabolites of the above four groups reveals that carnosine is a shared differentially metabolite in both human and mouse seminal plasma and dihydrotestosterone is a shared differentially metabolite in both testis and seminal plasma of mouse (Figure 4C), suggesting that oxidative damage and hormonal imbalance may contribute to reproductive damage caused by PTFE exposure.

Further functional enrichment analysis of these differential metabolites indicates that hormone metabolism was the most significantly enriched pathway in both species (Figure 4D,E). To verify the hormonal imbalance, we measured serum testosterone and dihydrotestosterone levels in mice and demonstrated that a gradual decrease in serum hormone levels as PTFE concentration increases (Figure 4F,G). Given that testicular leydig cells are primarily responsible for androgen production, we test the androgen secretion of TM3 (a mouse testicular leydig celline) in vitro. PTFE exposure directly downregulates androgen secretion in PTFE exposure group compared to control group (Figure 4H,I), which is consistent with the in vivo findings. In conclusion, these results suggest that PTFE exposure disrupts androgen secretion in testicular leydig cells, potentially contributing to male reproductive toxicity.

### 2.5 PTFE Exposure Induces Apoptosis in Germ Cells and Leydig Cells

**Figure 5:**
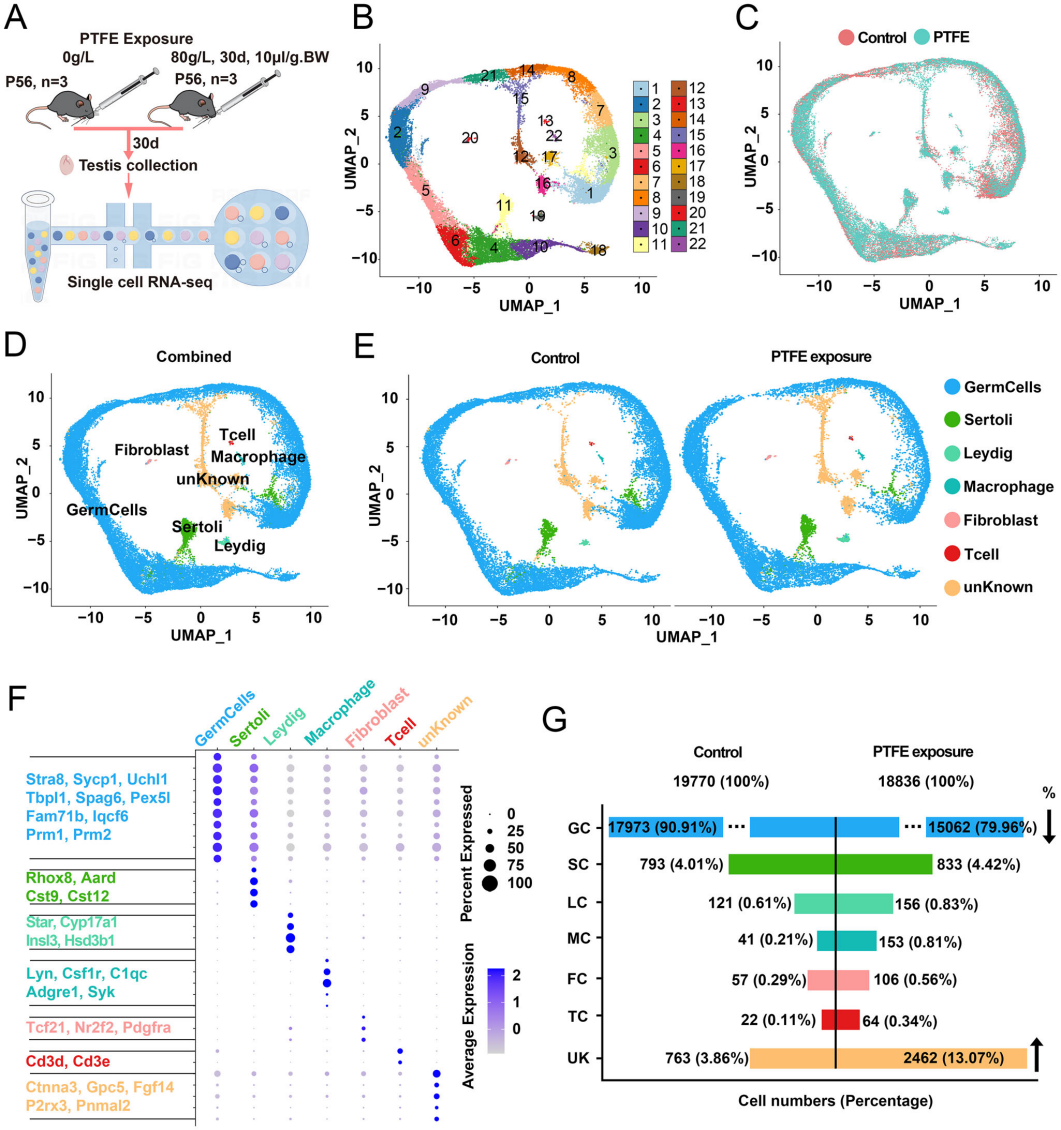
Single‐cell transcriptome analysis from control and PTFE‐exposed mouse testis. A) Flowchart overview of the experimental design for single‐cell RNA sequencing. B) UMAP (Uniform Manifold Approximation and Projection) plot illustrating the clustering of testicular cells from both control and PTFE‐exposed mouse samples, which reveals 22 distinct biological subtypes. C) UMAP plot presenting the clustering analysis of combined single‐cell transcriptome data from control and PTFE‐exposed mouse testicular cells, with clusters color‐coded and labeled. D,E) UMAP plots highlighting seven clusters from individual libraries of testicular cells isolated from control and 80 g L^−1^ PTFE‐exposed mouse testis. F) Dot plot depicting the expression of canonical marker genes in cells from control and PTFE‐exposed mouse testis. G) Symmetrical bar plot illustrating the quantity and percentage of various cell types in both control and PTFE‐exposed samples, with cell counts and percentages clearly labeled. Up and down arrows indicate cell percentage changes.

To examine the impact of PTFE exposure on spermatogenesis at the RNA level, single‐cell sequencing was performed (**Figure** **5**A). A total of 22 clusters were identified from the testicular tissues in both the control and PTFE‐exposed groups (Figure 5B), with cell distribution visualized in both groups (Figure 5C). The cells were annotated into six distinct populations (Figure 5D,E), with gene expression patterns depicted in bubble maps and feature plots (Figure 5F; Figure S4, Supporting Information). Cell proportion analysis revealed a significant loss of germ cells in the PTFE‐exposed group, accompanied by an increase proportion of unknown cell Figure 5G). To explore the underlying mechanisms, Gene Ontology (GO) enrichment analysis indicated that differentially expressed genes were significantly enriched in functions related to germ cell “apoptosis” and “differentiation.” In leydig cells, biological processes associated with “apoptosis” and “hormone metabolism” were also enriched (Figure S5A,B, Supporting Information). Additionally, single‐cell analysis showed a significant increase in the proportion of apoptotic cells in germ cells, leydig cells, sertoli cells, and unidentified cell populations (Figure S5C,D, Supporting Information). Also the expression of apoptotic genes, including *Bax* and *Caspase‐3*, was significantly elevated in both germ cells and leydig cells (Figure S5E,F, Supporting Information). In vitro and vivo experiments demonstrated that PTFE exposure induced apoptosis in GC‐1 (mouse spermatogonia cell line), GC‐2 (mouse spermatocyte cell line), TM3 (mouse leydig cell line), and SOX9‐positive sertoli cells decrease (Figure S5G–K, Supporting Information).

**Figure 6:**
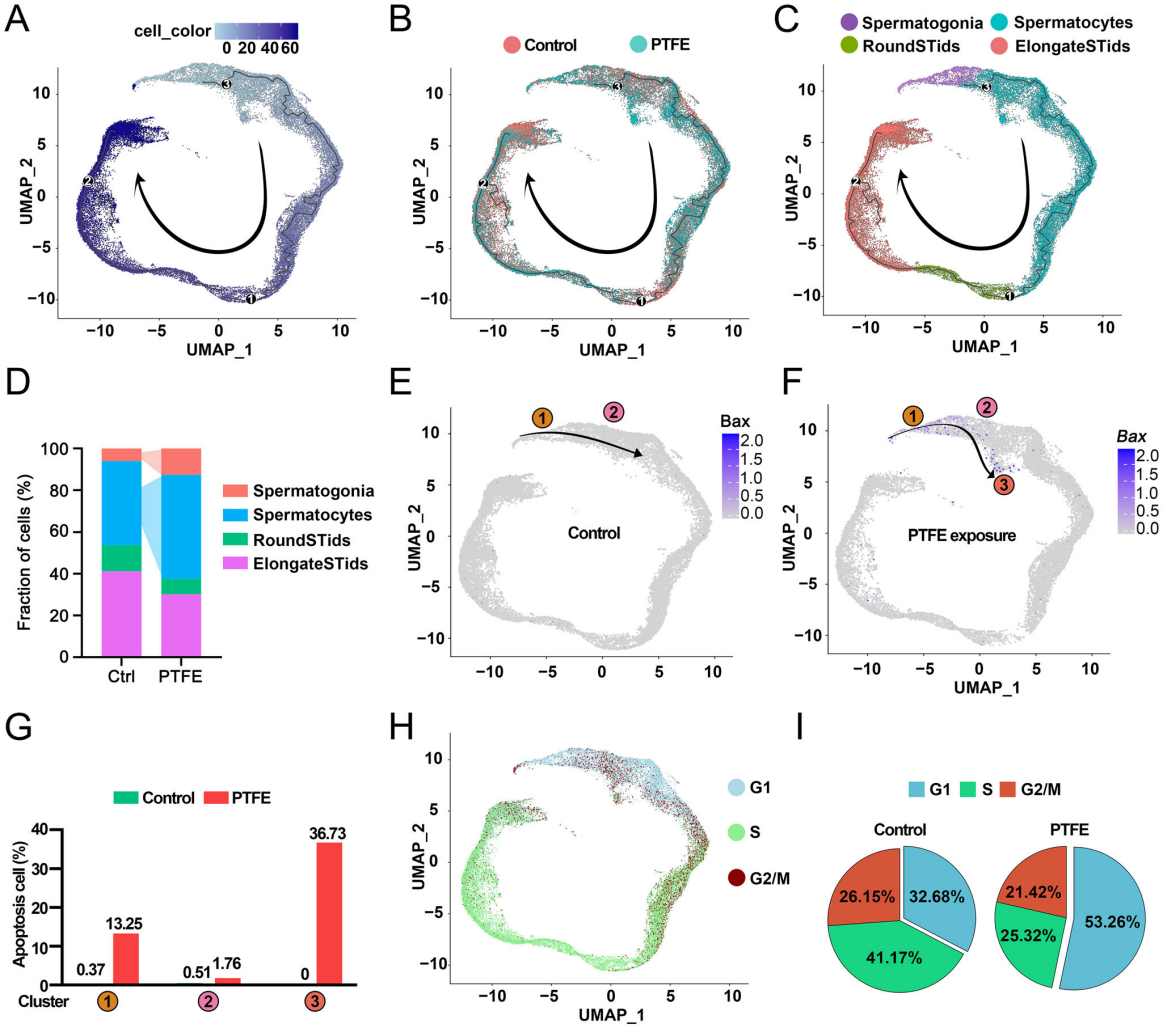
Comparison of germ cell development between control and PTFE exposure. A–C) Pseudotime analysis of control and PTFE‐exposed mouse germ cells, illustrating the developmental trajectory of spermatogonia (SPG), spermatocytes (SPC), round spermatids (RST), and elongate spermatids (EST). The black arrows indicate the direction of development. D) Proportions of SPG, SPC, RST, and EST in control and PTFE‐exposed mouse samples. E,F) Apoptosis analysis based on *Bax* expression levels in the UMAP‐defined single‐cell RNA sequencing (scRNA‐seq) transcriptomes of control and PTFE‐exposed mice. G) Quantification of apoptotic germ cells in control and PTFE‐exposed mice. H) UMAP plot displaying cell‐cycle phase analysis of germ cells. I) Quantification of cells in each cell‐cycle phase for control and PTFE‐exposed mice.

To further characterize apoptosis in the germ cells, subsets were annotated as spermatogonia, spermatocytes, round spermatids, and elongate spermatids (Figure S6A, Supporting Information). UMAP analysis revealed the distribution of cells in both control and PTFE‐exposed groups (Figure S6B,C, Supporting Information), and violin plots (Figure S6D, Supporting Information) and heat maps (Figure S6E, Supporting Information) provided gene annotations for the different cell types. GO enrichment analysis of differentially expressed genes (DEGs) revealed aberrant expression of genes enriched in the “apoptosis” in spermatogonia and “DNA double‐strand break (DSB) repair” in spermatocytes (Figure S6F, Supporting Information). This suggesting that PTFE exposure primarily induces apoptosis and differentiation abnormalities in spermatogonia and spermatocytes. Pseudotemporal analysis (**Figure** **6**A–C) and cell proportion analysis (Figure 6D) further demonstrated an increased percentage of spermatogonia and spermatocytes in the PTFE‐exposed group. Additionally, the apoptotic gene *Bax* was found to be highly expressed in both spermatogonia and primary spermatocytes (Figure 6E,F), reinforcing the role of apoptosis in germ cell loss.

Intriguingly, PTFE exposure resulted in the emergence of abnormal cell populations (cluster 3), with a significant increase in the proportion of apoptotic cells in this cluster (36.73%) compared to the control group (0%) (Figure 6G). These findings indicate that PTFE exposure induces apoptosis and disrupts the development of spermatogonia and spermatocytes. Furthermore, cell cycle analysis revealed a significantly higher proportion of cells in the G1 phase in the PTFE‐exposed group (53.26%) compared to the control group (32.68%) (Figure 6H,I), suggesting that these cells were transiently stagnant. Immunofluorescence staining further confirmed the increased presence of spermatogonia and spermatocytes in the PTFE‐exposed testis (Figure S7A–D, Supporting Information). Also the expression of apoptosis‐related genes (*Bax*, *Caspase‐3*), proliferation genes (*Ki67*), and cell cycle‐related genes (*Slx4*, *Vrk1*) was downregulated in spermatogonia and spermatocytes (Figure S7E–I, Supporting Information). Correspondingly, PAS and TUNEL staining of testicular sections demonstrated that PTFE exposure caused apoptosis of germ cells and leydig cells located outside the tubules (Figure S8A–E, Supporting Information). To explore the targets by which PTFE affects spermatocytes and leydig cells, our molecular docking results indicate that PTFE can directly target *Brca1*, *Smc5*, *Cyp11a1*, and *Cyp17a1* (Figure S8F, Supporting Information), supporting that PTFE exposure disrupts the homeostasis of proliferation and differentiation in spermatocytes and leydig cells, and eventually lead to cell apoptosis.

### 2.6 Disruption of Male Meiotic Processes by PTFE Exposure

Meiosis is a unique event during gametogenesis, playing a crucial role in genetic recombination and the maintenance of genomic integrity in germ cells.^[^ ^23^ ^]^ To evaluate the impact of PTFE exposure on meiotic spermatocytes, we conducted chromosome spreading analyses to investigate meiotic processes. In the control group, SYCP1, a marker of synapsis, was localized to the pseudoautosomal region (PAR) of XY chromosomes in pachytene spermatocytes, whereas in the PTFE‐exposed group, asynaptic behavior of autosomes was observed in pachytene spermatocyte (32.66% in the PTFE group vs. 8.80% in the control group). (Figure S9A,B, Supporting Information). Furthermore, HORMAD proteins (HORMAD1 and HORMAD2), which are indicative of unsynapsed or desynapsed chromosome axes, were utilized to assess meiotic progression. In control pachytene spermatocytes, ≈90.56% of autosome exhibited normal synapsis however, 31.79% of autosome manifests a higher frequency of autosomal asynapsis and synaptic defects at PTFE‐exposed pachytene spermatocytes (Figure S9C,D, Supporting Information), indicating that PTFE exposure disrupts chromosome synapsis.

The initial wave of γH2AX formation occurs during the leptotene stage, which is mediated by ATM following the occurrence of double‐strand breaks (DSBs), and this signal dissipates during the repair process of DSBs.^[^ ^24^ ^]^ A subsequent wave of γH2AX mediated by ATR emerges during zygotene and is associated with unsynapsed or unrepaired chromosomes.^[^ ^25^ ^]^ In both the control and PTFE‐exposed spermatocytes, γH2AX signals were observed during the leptotene and zygotene stages, indicating that the formation of DSBs is relatively normal in PTFE‐exposed spermatocytes (Figure S9E, Supporting Information). Upon completion of autosome synapsis at the onset of pachytene, DNA damage response (DDR) factors such as γH2AX, MDC1, and pATR typically diminish from autosomes and accumulate on sex chromosomes, thereby inducing meiotic silencing of unsynapsed chromatin (MSUC), called meiotic sex chromosome inactivation (MSCI).^[^ ^24^ ^]^ To investigate whether synaptic defects were associated with MSUC in spermatocytes exposed to polytetrafluoroethylene (PTFE), we conducted meiotic chromosome spreads and co‐staining γH2AX and SYCP3. In control pachytene spermatocytes, γH2AX signals were eliminated from autosomes and exhibited typical γH2AX localization on the XY body, indicating the completion of DSB repair. Conversely, the majority (13.07%) of PTFE‐exposed pachytene spermatocytes displayed persistent γH2AX signals on autosomes, suggesting that DNA damage response (DDR) remained active in synapsed homologs (Figure S9E,F, Supporting Information). In addition, developmental retardation was observed in PTFE‐exposed spermatocytes, characterized by a lower proportion of pachytene and diplotene spermatocytes compared to controls, indicating a delay in progression from pachytene to diplotene (Figure S9F, Supporting Information).

MDC1 is a γH2AX‐binding protein that mediates the chromosome‐wide spreading of γH2AX on the XY body.^[^ ^25^ ^]^ MDC1 mislocalization was significantly higher in PTFE pachynema (30.49%) compared to controls (11.74%) (Figure S9G,H, Supporting Information), hinting that the failure of chromosome‐wide spreading of γH2AX. Additionally, phospho‐S428 ATR (*P*‐ATR), the active form of ATR, failed to diffuse into XY chromatin and retained on autosomes in PTFE‐exposed spermatocytes (Figure S9I,J, Supporting Information). Collectively, these findings suggest that PTFE exposure disrupts the normal elimination of γH2AX from autosomes and impairs the DNA damage response during meiosis, leading to defective synapsis and meiotic progression delays.

### 2.7 PTFE Targets SKAP2 Damaging Spermiogenesis

**Figure 7:**
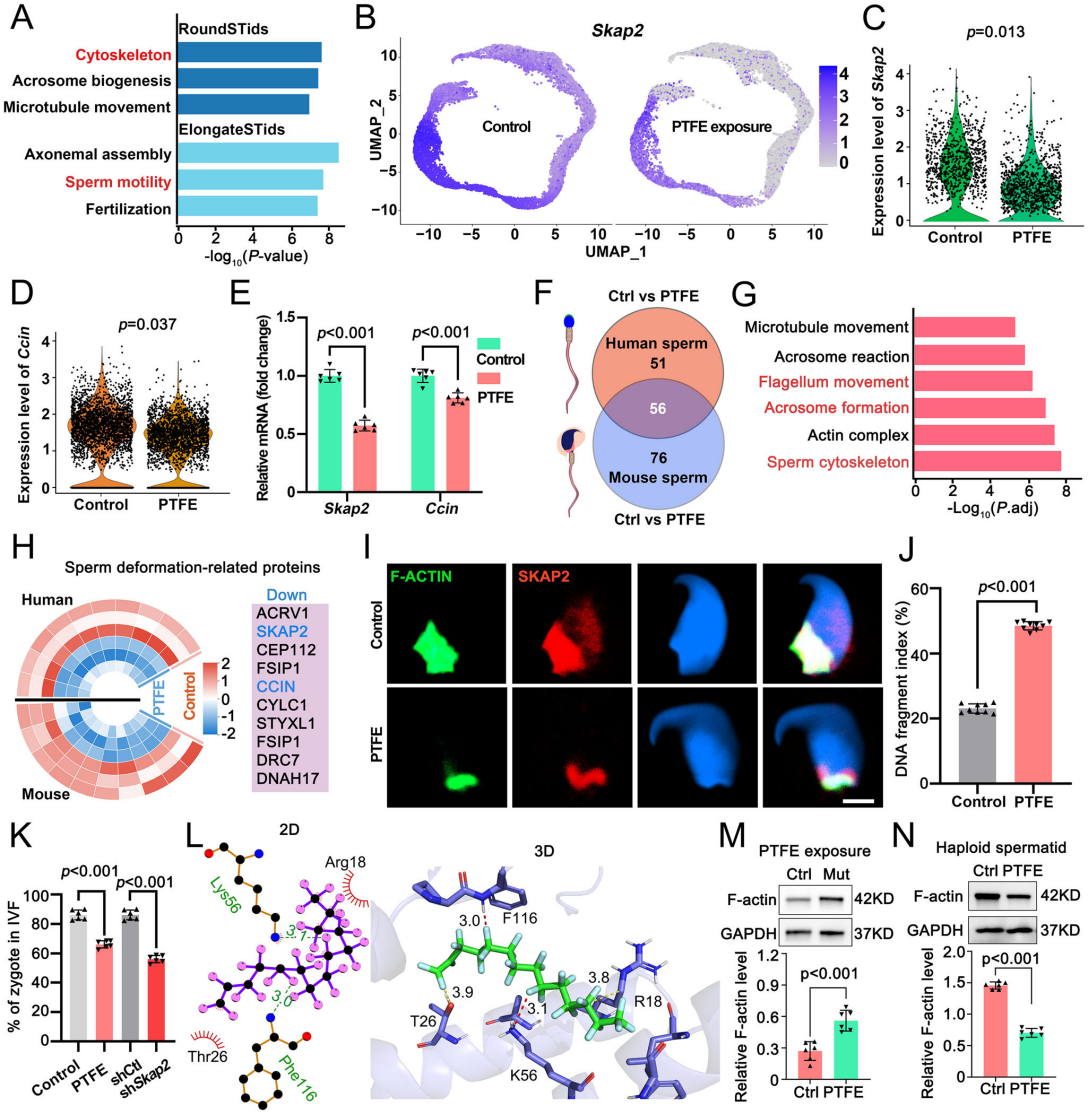
PTFE targets SKAP2 disrupting spermiogenesis process. A) Gene Ontology (GO) analysis indicated that differentially expressed genes (DEGs) in the haploid spermatids (Round STids and Elongate STids) clusters were significantly enriched for cytoskeletal function and sperm motility, respectively. B) UMAP plots visualize *Skap2* expression pattern in control and 80 g L^−1^ PTFE‐exposed mouse germ cells. C,D) Violin plots illustrate the expression patterns of variable genes, *Skap2* (C) and *Ccin* (D), within haploid spermatid clusters. E) RT‐qPCR analysis demonstrated a significant reduction in mRNA expression levels of *Skap2* and *Ccin* in haploid spermatids. Data are presented as mean ± SD, with *p*‐values calculated using a two‐sided Student's *t*‐test. F) Venn diagram depicts the overlap of differentially expressed proteins between human and mouse spermatozoa from control or PTFE exposure. Three mice were subjected to 80 g L^−1^ PTFE exposure, and three human sperm samples were randomly selected from the PTFE‐exposed group. G) GO term analysis of the 56 overlapping proteins identified in (F) is presented. H) A circle heatmap and list display the overlap of differentially expressed proteins related to spermiogenesis in both human and mouse sperm. The color represents the relative protein expression. The digital label on the color bar indicates the specific change trend. I) Immunofluorescence staining of the cytoskeletal marker F‐ACTIN (green) and SKAP2 (red) in control and PTFE‐exposed sperm is shown. Scale bar = 5 µm. J) Quantification of the Sperm DNA Fragmentation Index (SDFI) is presented. SDFI refers to the percentage of sperm with broken DNA strands to all sperm, which can be used to evaluate the integrity of sperm DNA. Data are expressed as mean ± SD, with *p*‐values calculated using a two‐sided Student's *t*‐test. K) Quantification of the zygote percentage after IVF is presented. Data are expressed as mean ± SD, with *p*‐values calculated using a two‐sided Student's *t*‐test. L) Docking model of PTFE molecules with the SKAP2 protein. In the 2D interaction diagram (left), the Van der Waals and hydrophobic interactions are indicated by eyelash‐like symbols, and hydrogen bonds are represented by green dotted lines. The 3D structural diagram (right) shows the hydrogen bonds as red dotted lines, and van der Waals interactions as yellow dotted lines. M) Protein expression analysis in 293T cells. The expression levels of SKAP2 were evaluated in both wild‐type and mutant (Thr26 mutation in the homodimerization region) 293T cells after treatment with 12.5 mg L^−1^ PTFE for 12 h. Data are presented as mean ± SD. Statistical significance was determined using a two‐sided Student's *t*‐test. N) Protein expression levels were measured in sorted haploid spermatids from the testes of control and PTFE‐treated (12.5 mg L^−1^ for 12 h) groups. Data are presented as mean ± SD, and *p*‐values were calculated using a two‐sided Student's *t*‐test.

During spermiogenesis, haploid spermatids undergo a series of crucial events including acrosome formation, nuclear condensation, and flagellum elongation, which are collectively regulated by a group of genes known as spermiogenesis genes.^[^ ^26^ ^]^ In this study, single‐cell RNA sequencing revealed a significant differential expression of genes associated with the cytoskeleton and motility in both round and elongate spermatids (**Figure** **7**A) suggesting that PTFE exposure may influence spermiogenesis by affecting the formation of the sperm cytoskeleton. Further analysis identified two genes, *Skap2* and *Ccin*, that were significantly downregulated in haploid spermatids exposed to PTFE (Figure 7B–E). To examine changes in protein levels in mature sperm following PTFE exposure, we analyzed the protein profiles in the epididymal sperm of both control and PTFE‐exposed groups. This analysis revealed 107 differentially expressed proteins in humans and 132 in mice (Figure 7F), with 56 overlapping differential proteins identified across both species. GO enrichment analysis indicated that these proteins were primarily involved in the sperm cytoskeleton, acrosome formation, and flagellar movement (Figure 7G). Among these proteins, SKAP2 and CCIN attract our attention due to their association with spermiogenesis (Figure 7H).^[^ ^27^ ^]^ Immunofluorescence staining revealed that in control sperm, SKAP2 co‐localizes with F‐ACTIN on the lower side of the sperm head, while PTFE exposure disrupted the normal localization of both F‐ACTIN and SKAP2 (Figure 7I), suggesting that the abnormal localization of F‐ACTIN and SKAP2 in sperm may be involved in PTFE‐induced sperm malformations.

To further explore the physiological role of *Skap2* during spermiogenesis and male fertility, we injected shRNA‐*Skap2* into the seminiferous tubules via the efferent ductules and generated *Skap2* knockdown (KD) mouse testes (Figure S10A, Supporting Information). After 7 days, both mRNA and protein levels of *Skap2* were significantly reduced (Figure S10B–D, Supporting Information). Periodic acid‐Schiff (PAS) staining revealed the normal tubules, while CASA analysis indicated a significant reduction in sperm motility and increase of deformed spermatozoa in the sh*Skap2* group compared to the shControl group (Figure S10E–I, Supporting Information), which is resembled to PTFE‐induced abnormal spermiogenesis. Further analyses revealed that elevated rates of DNA fragmentation were detected in both PTFE‐exposed and SKAP2 knockdown mice (Figure 7J; Figure S10J,K, Supporting Information). In addition, in vitro IVF experiments indicated that both PTFE exposure and SKAP2 knockdown significantly reduced fertilization rates in mice (Figure 7K). Fertility test showed that knock down of SKAP2 caused decrease of average pups per litter (Figure S10L, Supporting Information). Strikingly, acrosome co‐localization of SKAP2 with PNA was observed, which supported the unique function of SKAP2 on haploid spermatids (Figure S10M, Supporting Information). To further confirm that PTFE directly targets SKAP2, we conducted advanced molecular docking analyses. The docking score between PTFE and the SKAP2 protein was –7.062 kcal mol^−1^, indicating a strong binding affinity (scores below –7 kcal mol^−1^ typically reflect strong interactions). PTFE exhibited specific binding sites on the SKAP2 protein, forming two hydrogen bonds with the NH3‐Lys56 and the NH‐Phe116, at distances of 3.1 Å and 3.0 Å, respectively. Additionally, van der Waals interactions were observed between PTFE and surrounding residues Arg18 and Thr26 (Figure 7L). These findings suggest that PTFE binds strongly to amino acid residues Arg18, Thr26, and Lys56 within the Homodimerization region, as well as Phe116 within the PH domain of SKAP2. This interaction was further supported by in vitro mutagenesis experiments in cell lines. Specifically, mutation of Thr26 significantly attenuated PTFE‐induced impairment of SKAP2‐mediated F‐actin regulation (Figure 7M), underscoring PTFE's specificity for the Homodimerization region. To determine whether PTFE acts directly on haploid spermatids rather than indirectly through spermatogonia or spermatocytes, we isolated haploid spermatids from mouse testes. Upon in vitro PTFE exposure, these cells showed a marked reduction in F‐actin levels, confirming a direct effect (Figure 7N). Collectively, these results provide compelling evidence that PTFE induces asthenoteratozoospermia by directly targeting the Homodimerization domain of SKAP2 in haploid spermatids.

### 2.8 Extracellular Vesicles‐SKAP2 Improves Sperm Quality in Both Humans and Mice

**Figure 8:**
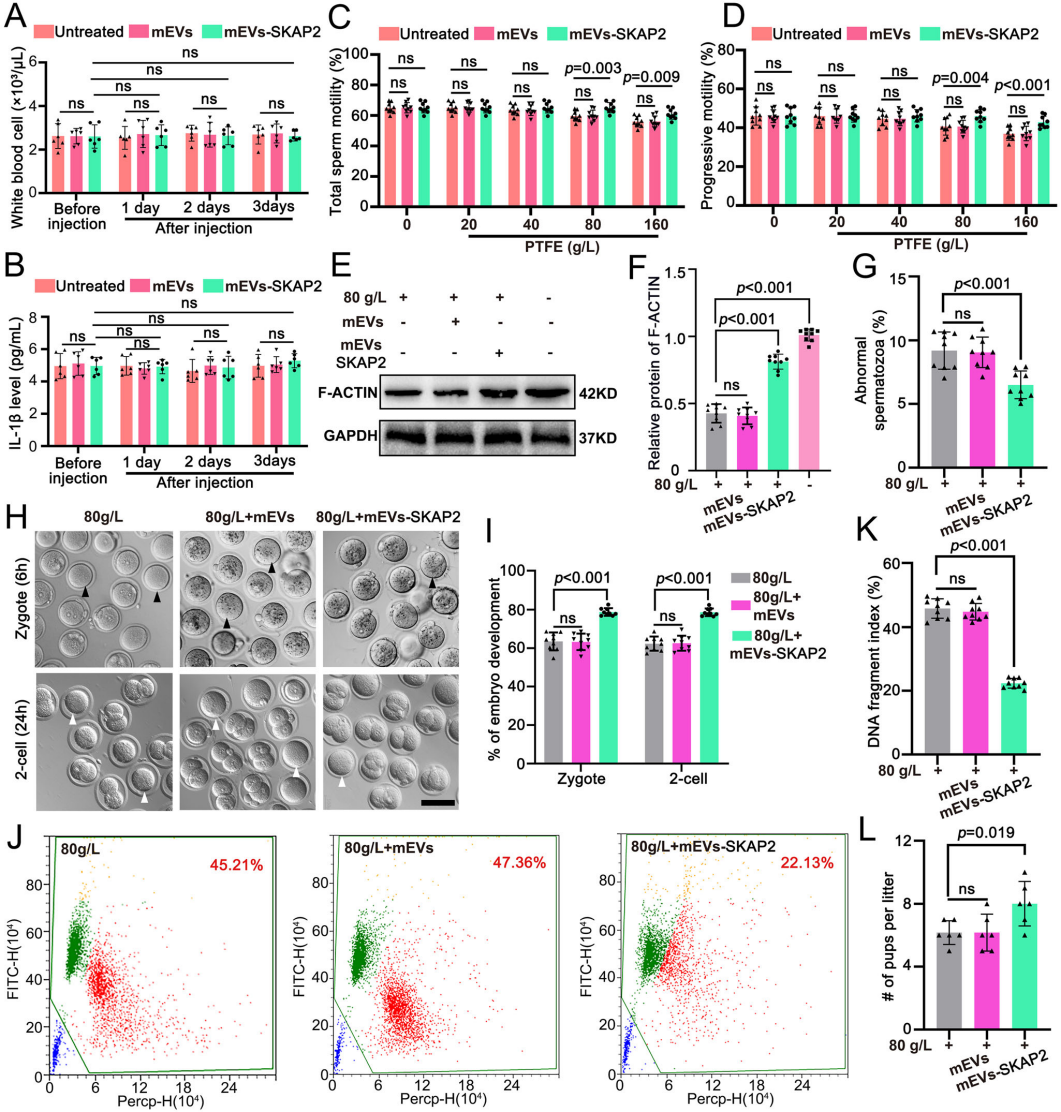
Extracellular vesicles‐SKAP2 rescues PTFE‐induced decline of sperm quality in mice. A) White blood cell counts in mouse peripheral blood were measured using an automated hematology analyzer following tail vein collection. Data are expressed as mean ± SD. “NS” denotes no statistically significant difference. *p*‐values were calculated using a two‐tailed Student's *t*‐test. B) Serum IL‐1β levels in mice were quantified using enzyme‐linked immunosorbent assay (ELISA). Data are presented as mean ± SD. “NS” indicates no statistical significance. *p*‐values were determined using a two‐tailed Student's *t*‐test. C,D) In vivo injection via the efferent ducts three days, CASA analysis was conducted to assess total sperm motility (C) and progressive motility (D) across groups, untreated, mEVs, and mEVs‐SKAP2, after exposure to different PTFE doses (0, 20, 40, 80, and 160 g L^−1^) over a 30‐day period. Data are presented as mean ± SD. NS indicates no significance. *p*‐values were determined using a two‐sided Student's *t*‐test. E) Representative western blot showing the protein abundance of F‐ACTIN in spermatozoa following in vivo injection of mEVs or mEVs‐SKAP2. GAPDH serves as a loading control. F) Quantitative analyses of the protein expression level of F‐ACTIN in panel (E). Data are presented as mean ± SD. *p* values (Student *t*‐test, two‐sided). G) Histogram shows the proportion of abnormal spermatozoa from untreated, mEVs, and mEVs‐SKAP2 groups after injection. 100 spermatozoa were counted for each mouse. Data are presented as mean ± SD. *p* values (Student *t*‐test, two‐sided). H) Representative differential interference contrast (DIC) images of zygotes and 2‐cell embryos from the untreated, mEVs, and mEVs‐SKAP2 groups after injection are shown. A total of 9 female mice, along with 160 fertilized oocytes and 150 two‐cell stage embryos, were analyzed per group. With a scale bar of 100 µm. Black and white arrowheads indicated unfertilized oocytes in stage of zygote and 2‐cell, respectively. I) The quantification of zygote and 2‐cell embryo percentages following IVF, as depicted in (H), is expressed as mean ± SD. *p*‐values were calculated using a two‐sided Student's *t*‐test. J) Flow cytometry analysis was performed to evaluate sperm DNA fragmentation across the untreated, mEVs, and mEVs‐SKAP2 groups after injection. K) Statistical analysis of DNA fragment index from the untreated, mEVs, and mEVs‐SKAP2 groups in panel (J). Data are presented as mean ± SD. *p* values (Student *t*‐test, two‐sided). L) Fertility test was conducted to determine the number of pups per litter among the untreated, mEVs, and mEVs‐SKAP2 groups. Following three days of extracellular vesicles‐SKAP2 injection via the efferent ductules, mating, vaginal plug checks, and pup counts were performed. Data are expressed as mean ± SD, with *p*‐values calculated using a two‐sided Student's *t*‐test.

Extracellular vesicles (EVs) are membrane‐enclosed structures and could serve as carriers for biologically active molecules such as proteins, lipids, and genetic material.^[^ ^28^ ^]^ In this study, we screened that SKAP2 is a key downstream protein induced by PTFE exposure during spermatogenesis. To determine whether extracellular vesicles‐based therapy could rescue sperm quality in mice exposed to PTFE, we developed extracellular vesicles containing SKAP2 (mEVs‐SKAP2) and injected them into the efferent ducts of the testis in vivo and co‐incubated them with sperm in vitro (Figure S11A, Supporting Information). First, we assessed whether mEVs‐SKAP2 elicited an immune response in mice. By measuring white blood cell counts and serum IL‐1β levels, we found that microinjection of mEVs‐SKAP2 into the seminiferous tubules did not induce a significant immune reaction (**Figure** **8**A,B). These results support the feasibility of further investigating the functional role of mEVs‐SKAP2 in vivo. Strikingly, CASA demonstrated that mEVs‐SKAP2 significantly rendered the sperm capable of improved motility, compared to untreated and mEVs treated group (Figure 8C,D; Figure S11B,C, Supporting Information). Importantly, Compared with the three‐day treatment, the seven‐day protocol yielded better results, particularly at the 80 g L^−1^ concentration, overall sperm motility increased from 65.14% to 67.58%, and progressive motility improved from 46.09% to 49.52% (Figure 8C,D; Figure S11D,E, Supporting Information).

The F‐actin is essential for sperm morphology and motility. Therefore, to assess the impacts of mEVs‐SKAP2 on F‐ACTIN assembly, we measured and revealed that increased F‐ACTIN levels in the testes of PTFE‐exposed mice treated with mEVs‐SKAP2 in comparison with untreated and mEVs (Figure 8E,F). However, we did not observe significant improvements in the diameters of seminiferous tubules and in the presence of vacuolar tubules after injection of mEVs‐SKAP2 (Figure S11F,G, Supporting Information). It is worth noting that compared with in vitro co‐incubation, the method of injecting into the efferent ductules has a more obvious effect in improving malformed sperm (Figure 8G; Figure S11H, Supporting Information). Sperm morphology and motility can contribute to the fertilization ability. Thus, further in vitro fertilization (IVF) assays were conducted using spermatozoa obtained from the caudal epididymis of male mice exposed to PTFE, along with oocytes harvested from female wild‐type (WT) mice. The results demonstrated that mEVs‐SKAP2 significantly increased fertilization rates and two‐cell embryo development in vivo in comparison with mEVs (Figure 8H,I). Additionally, mEVs‐SKAP2 significantly reduced DNA fragmentation and improved the fertility in comparison with mEVs (Figure 8J–L). In conclusion, these results indicate that the treatment with mEXO‐SKAP2 can improve the decline in sperm quality and the reduction in fertilization rate caused by polytetrafluoroethylene (PTFE) exposure.

**Figure 9:**
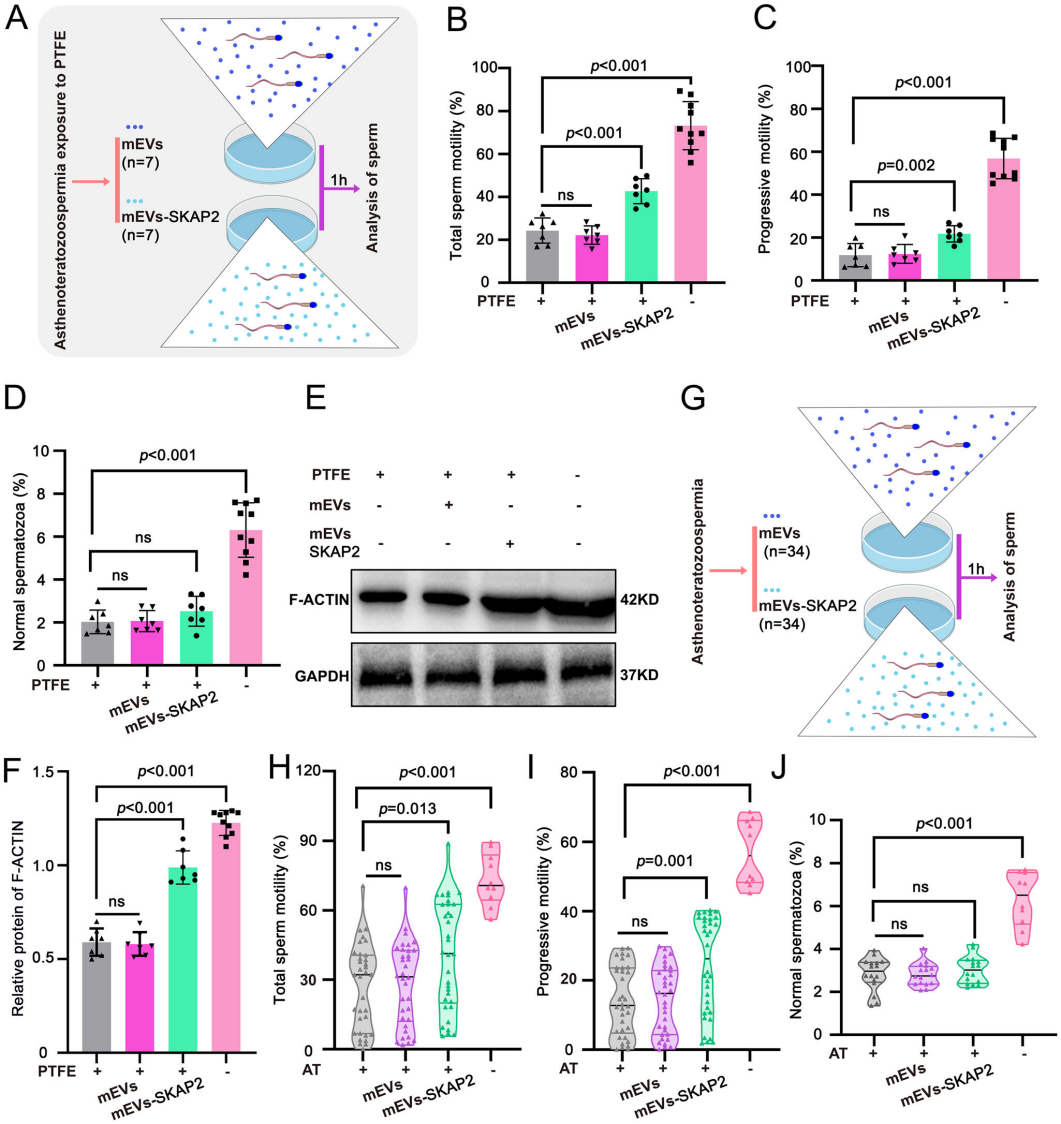
mEVs‐SKAP2 repairs asthenoteratozoospermia in humans. A) The diagram depicts the co‐incubation of mEVs or mEVs‐SKAP2 with spermatozoa from individuals exposure to PTFE. Sperm (2.5 × 10⁶ per well) were cultured in 1 mL BWW medium at 37°C with 5% CO₂ for 1 h. Following co‐incubation, sperm samples were collected for subsequent analysis. B,C) The CASA analysis of total sperm motility (B) and progressive motility (C) was conducted following in vitro co‐incubation of mEVs or mEVs‐SKAP2 with sperm from 7 patients diagnosed with asthenoteratozoospermia exposure to PTFE. Data are expressed as mean ± SD. NS, not significant. *p*‐values were calculated using Student's *t*‐test (two‐sided). D) The percentage of sperm exhibiting normal morphology was assessed following in vitro co‐incubation of mEVs or mEVs‐SKAP2 with sperm from 7 patients with asthenoteratozoospermia. A total of 100 spermatozoa were counted for each individual. Data are expressed as mean ± SD. NS, not significant. *p*‐values were calculated using Student's *t*‐test (two‐sided). E) Representative western blot showing the protein abundance of SKAP2 and F‐ACTIN in sperm of asthenoteratozoospermia following in vitro co‐incubation of mEVs or mEVs‐SKAP2. GAPDH serves as a loading control. F) Quantitative analyses of the protein expression level of F‐ACTIN in panel (E). Data are presented as mean ± SD. *p* values (Student *t*‐test, two‐sided). G) A schematic illustration of the co‐incubation of mEVs or mEVs‐SKAP2 with sperm from asthenoteratozoospermia (AT). Sperm (2.5 × 10⁶ per well) were cultured in 1 mL BWW medium at 37°C with 5% CO₂ for 1 h. H,I) CASA analysis of total sperm motility (H) and progressive motility (I) following in vitro co‐incubation of mEVs or mEVs‐SKAP2 with sperm from 34 patients with asthenoteratozoospermia (AT). Data are expressed as mean ± SD. NS, not significant. *p*‐values were calculated using Student's *t*‐test (two‐sided). J) The percentage of normal sperm comprising 16 patients following in vitro co‐incubation of mEVs or mEVs‐SKAP2 with sperm from asthenoteratozoospermia. 100 spermatozoa were evaluated for each individuals. Data are expressed as mean ± SD. NS, not significant. *p*‐values were calculated using Student's *t*‐test (two‐sided).

Interestingly, our study found the mEXO‐SKAP2 can not only recovery sperm motility in PTFE exposure model but also in lead exposure model (Figure S11I and Table S3, Supporting Information), further supporting the therapeutic strategy of mEVs‐SKAP2 in asthenoteratozoospermia induced by environmental poisons. Subsequently, to assess the clinical relevance of mEVs‐SKAP2, we examined a cohort of seven patients exposed to PTFE and diagnosed with asthenoteratozoospermia. After incubation mEVs‐SKAP2 with human semen samples (**Figure** **9**A; Table S4, Supporting Information), sperm motility showed a significant increase (Figure 9B,C). However, sperm morphology did not show a significant improvement, which was consistent with mice following treatment with mEVs‐SKAP2 (Figure 9D; Figure S11H, Supporting Information). Mechanistically, consistent with the increased F‐ACTIN in mice following treatment with mEVs‐SKAP2, a significant increase of F‐ACTIN was observed in humans spermatozoa (Figure 9E,F). As the variety of adverse factors that contribute to asthenoteratozoospermia and limiting individuals' exposure to PTFE (*n* = 7), we further validated the therapeutic efficacy of mEVs‐SKAP2 in a larger cohort of asthenoteratozoospermia patients (*n* = 34) (Figure 9G; Table S4, Supporting Information). The results indicated that mEVs‐SKAP2 can also repair sperm motility of asthenoteratozoospermia individuals (Figure 9H,I), while no significant improvement in sperm morphology (Figure 9J). Collectively, these results suggest that mEXO‐SKAP2 modulates the formation of F‐ACTIN cytoskeleton and has a therapeutic potential for asthenoteratozoospermia.

## 3 Discussion

As the reproductive lifespan and fertility of male mammals are contingent upon sperm quality,^[^ ^29^ ^]^ we investigated the spermatogenesis at various stages and sperm parameters. Our findings indicate that PTFE exposure led to a decrease in sperm counts, motility, and normal morphology in both humans and mice. PTFE exposure delays in the development of spermatogonia and spermatocytes, as well as abnormal sperm acrosome formation. Single‐cell analyses demonstrated apoptosis in both germ and leydig cells following PTFE exposure, as evidenced by a reduction in the proliferative marker *Ki67* and an increase in apoptotic markers, including *Bax* and *Caspase‐3*. Furthermore, the downregulation of cell cycle genes *Slx4* and *Vrk1* reinforces the concept that PTFE serves as a potent environmental disruptor, impairing the differentiation of spermatogonia and spermatocytes, ultimately resulting in apoptotic process.

At the pachytene stage of meiosis, exposure to PTFE significantly disrupted homologous autosome synapsis, leading to abnormalities in the DNA damage response (DDR) and further compromising the differentiation of spermatocytes, as well as inducing apoptosis. The initiation of meiotic silencing on unsynapsed chromosomes requires DDR factors, such as γH2AX, which is a key component in meiotic silencing of unsynapsed chromosomes (MSCI).^[^ ^24^, ^30^ ^]^ Specifically, we observed that a substantial proportion of pachytene spermatocytes exposed to PTFE exhibited retained γH2AX signals on autosomes compared to the control group, indicating disrupted double‐strand break (DSB) repair processes. Consistently, Pujol et al. recently reported that exposure to polystyrene nanoplastics induces alterations in the expression of genes involved in the formation and repair progression of DNA double‐strand breaks.^[^ ^31^ ^]^ Notably, the phosphorylation of H2AX induced by unsynapsed chromosomes is catalyzed by the DDR factor ATR.^[^ ^32^ ^]^ During meiotic prophase I, abnormalities in the DDR pathway trigger meiotic arrest and subsequent cell elimination.^[^ ^25^, ^33^ ^]^ In the current study, we meticulously analyzed the abnormal localization of γH2AX at the pachytene stage, marking the unsynapsed autosomes and the XY body. Our data suggest that the retention of *p*‐ATR on autosomes is likely to contribute to H2AX phosphorylation in spermatocytes exposed to PTFE. In summary, our findings indicate a novel detrimental effect of PTFE on the regulation of the DDR pathway in mammalian spermatocytes, which will enhance our understanding of the damages caused by PTFE exposure during meiosis.

Spermiogenesis are required to undergo a remarkable transformation and sequential programmed transitions.^[^ ^34^, ^35^, ^36^ ^]^ To further dissect the underlying mechanisms mediating the effects of PTFE exposure on spermiogenesis in male mice, we conducted single‐cell transcriptomic sequencing of testicular tissue from mice at three months of age. Our analysis revealed significant alterations in gene expression in mice exposed to PTFE, with a notable down‐regulation of mRNA expression of *Ccin* and *Skap2* in haploid spermatids. Proteomic analyses further confirmed that PTFE exposure in both human and mouse models resulted in decreased expression of proteins such as SKAP2 and CCIN. Previous studies have demonstrated that the deletion of *Ccin* leads to sperm deformation in both humans and mice.^[^ ^27^, ^37^ ^]^ To explore the role of SKAP2 in PTFE‐induced sperm actin and cytoskeletal remodeling, we constructed an SKAP2‐specific shRNA, injected it into the efferent ductules, and successfully knocked down SKAP2 expression. This decreased SKAP2 resulted in abnormal F‐ACTIN and sperm malformation, consistent with PTFE‐exposure mouse model.

Given the adverse effects of polytetrafluoroethylene (PTFE) on spermatogenesis, there is an urgent need to develop therapeutic strategies to mitigate the impact on male reproductive health. One promising approach involves utilizing extracellular vesicles (EVs) to deliver targeted therapies aimed at restoring normal cellular function.^[^ ^38^ ^]^ They are linked to both the intrinsic cellular ‘state’ and the extracellular signals received from the environment, positioning them as an ideal platform for targeted therapeutic interventions.^[^ ^39^ ^]^ The testis is rich in extracellular vesicles that mediate signal transduction between tubule germ cells and leydig cells, promoting testicular development and spermatogenesis.^[^ ^40^, ^41^ ^]^ During spermiogenesis, extracellular vesicles (EVs) play a pivotal role in modulating sperm functions by interacting with developing spermatozoa through membrane fusion. This interaction facilitates the transfer of bioactive molecules such as proteins, lipids, and RNAs, which collectively enhance critical functional attributes. Specifically, EVs contribute to the remodeling of the sperm membrane and the delivery of signaling components that significantly improve sperm motility, capacitation, and the acrosome reaction.^[^ ^42^, ^43^, ^44^ ^]^ In this study, we leverage the natural targeting properties of extracellular vesicles to directly deliver SKAP2 to germ cells. Computer‐assisted sperm analysis (CASA) indicates improvements in sperm motility, both in vivo and in vitro. Notably, the in vivo results were more encouraging, with injection significantly enhancing sperm motility at 160 g L^−1^, while in vitro incubation showed no significant effect. Additionally, there was a decrease in the percentage of abnormal spermatozoa following in vivo efferent tubule injection, whereas no significant decrease was observed in vitro co‐incubation. These findings suggest that in vivo therapies utilizing extracellular vesicles‐SKAP2 improves spermatid remodeling in the testis and offer more substantial benefits for treating asthenoteratozoospermia compared to in vitro fertilization (IVF)‐based approaches.

To further clarify the extracellular vesicles‐mediated SKAP2 therapy holds significant promise to counteract PTFE‐induced malformed spermatozoas during spermiogenesis. We explore the aid in the restoration of sperm morphology during spermiogenesis and promoting the production of normal spermatozoa. Supplementation with extracellular vesicles (EVs) containing SKAP2 restores DNA integrity and promotes sperm cytoskeletal remodeling, as evidenced by enhanced F‐ACTIN assembly. This intervention not only mitigates DNA fragmentation but also facilitates proper chromatin packaging, crucial for maintaining genomic stability. The observed upregulation of F‐ACTIN underscores the reorganization of cytoskeletal architecture, which is essential for key spermiogenic events such as acrosome formation, nuclear shaping, and flagellar development. In addition, Reyes et al. reported that the process of actin polymerization is crucial for capacitation, as blocking it stops sperm from adhering to and fusing with the ovule.^[^ ^45^ ^]^ Consequently, the fertilization ability increased after IVF indicated that mEVs‐SKAP2 also facilitate F‐actin fromation and capacitation. In clinic, we explored the improvement potential of extracellular vesicles in cases of asthenoteratozoospermia and demonstrated the repair of sperm motility. Consistent with in mice spermatozoa, we verified the F‐ACTIN assembly in human sperm. Collectively, these data from both human and mouse models support the efficacy of extracellular vesicle (EV)‐mediated SKAP2 intervention.

Fertilization ability depends on normal sperm morphology, motility, capacitation, and acrosomal reactions.^[^ ^46^, ^47^ ^]^ Improved fertilization was observed after mEVs‐SKAP2 treatment in mouse model. We hope that clinical trials will be conducted in the future to explore whether the application of mEVs‐SKAP2 can improve fertility in assisted reproduction in asthenoteratozoospermia. However, several challenges must be addressed before this therapeutic strategy can be implemented in clinical practice. First, a thorough evaluation of the long‐term safety and intergenerational impacts is necessary, particularly regarding their effects on reproductive health. Second, future research should optimize methods used for the isolation and preparation of extracellular vesicles and establish the most effective dosing and administration strategies. Third, while SKAP2 shows promise as a therapeutic target in asthenoteratozoospermia by remodeling the sperm cytoskeleton, whether its supplementation will cause other effects on sperm still needs to be further studied. Finally, the interaction between PTFE and other environmental toxins warrants further exploration, as exposure to multiple chemicals may exacerbate reproductive dysfunction. Understanding these interactions will be key to developing more comprehensive therapeutic strategies.

In conclusion, this study illustrates in detail on the molecular mechanisms by which polytetrafluoroethylene (PTFE) affects spermatogenesis and male fertility. More importantly, we propose a promising therapeutic strategy that uses extracellular vesicles to deliver SKAP2 to restore the damage caused by environmental toxins. Our study will lay the foundation for the development of novel treatment methods for male infertility, particularly for individuals exposed to environmental pollutants.

## 4 Experimental Section

### Human Semen and Urine Sample Collection and Analysis

To assess the regional impact of PTFE exposure, male participants were recruited from couples seeking care at reproductive medicine centers across various locations, adhering to the inclusion and exclusion criteria established in our previous study.^[^ ^7^ ^]^ Semen and urine samples were collected from the following sites, Henan People's Hospital in Zhengzhou (Site 1) from November 1 to November 28, 2023, Maternal and Child Health Care Hospital of Shandong Province in Jinan (Site 2) from January 2 to January 15, 2024, and from March 15 to March 29, 2024, Maternal and Child Health Care Hospital of Xiaogan in Xiaogan, Hubei Province (Site 3) from January 8 to February 2, 2024, and the Fourth Affiliated Hospital of School of Medicine in Yiwu, Zhejiang Province (Site 4) from January 1 to March 1, 2025. Additionally, for the extracellular vesicles‐SKAP2 intervention treatment, further samples were collected from reproductive centers of five regions in Chongqing, hangzhou, Kunming, Zhengzhou, and Yiwu. Semen samples were collected through masturbation after a sexual abstinence period of 2 to 5 days and were subsequently analyzed following liquefaction at 37°C for 30 min. In the laboratory, semen analysis encompasses tests for sperm concentration, motility, morphology, pH, and liquefaction time, all of which provide essential insights into sperm health. For the analysis of sperm morphology, slides were stained using Diff‐Quik (Funuo). This process was conducted in accordance with the Sixth Edition of the World Health Organization (WHO) Laboratory Manual for the Examination and Processing of Human Semen. Semen quality was classified as ‘normal’ or ‘abnormal’ based on the following reference values (5th percentile), progressive motility (< 30% motile sperm), total motility (progressive + non‐progressive motility) < 42%, normal spermatozoa (< 4%), total sperm count (< 39 million), semen volume (< 1.4 mL), and sperm concentration (< 16 million mL^−1^). Data are presented as mean ± SD. All procedures adhered to the ethical guidelines outlined in the World Medical Association's Declaration of Helsinki. The study received approval from the Institutional Ethics Committee of the Obstetrics and Gynecology Hospital, Fudan University, and informed consent was obtained from all participants (Approval NO., kyy2024‐280).

### Mice

All animal experiments conducted in this study adhered to the ethical guidelines and regulations established by relevant institutional and national authorities. The procedures were performed in accordance with the guidelines set forth by the Institutional Animal Care and Use Committee (IACUC) of Zhejiang University, China (Approval NO., ZJU20250130). Efforts were made to minimize animal suffering and the number of animals used in alignment with the 3Rs principle, Replacement, Reduction, and Refinement. Mice were housed in a pathogen‐free facility at the Laboratory Animal Center, International Institutes of Medicine, fourth Affiliated Hospital of the School of Medicine. The study was designed to ensure the ethical treatment of animals while achieving the scientific objectives. For mouse model, according to the previous study,^[^ ^48^ ^]^ the administration of corn oil for the control group and PTFE microplastics suspended in corn oil for microplastic doses were performed.

### Epididymal Sperm Staining and Analysis in Mice

The caudal epididymis was carefully dissected from 8‐week‐old male mice. To release sperm, the epididymis was minced and incubated in pre‐warmed PBS for 10–20 min at 37°C. For sperm morphology analysis, slides were stained with Hematoxylin and Eosin (H&E) (Servicebio, G1076) following the manufacturer's instructions. Computer‐assisted sperm analysis (CASA) was conducted to evaluate sperm concentration and motility. The percentage of abnormal sperm was determined by examining at least 100 spermatozoa from each mouse.

### Histological Analysis in Mice

The testes and epididymides were carefully dissected from mice and fixed overnight in Bouin's solution (Sigma, HT10132) at 4°C to preserve their cellular structure and prevent degradation. After fixation and dehydration, the tissues were embedded in paraffin and sectioned into 5 µm‐thick slices. Following deparaffinization and rehydration, the tissue sections were stained with either hematoxylin and eosin (H&E) or periodic acid‐Schiff (PAS) to highlight different components of the tissue, including cell nuclei and cytoplasm, in accordance with the manufacturer's instructions. The stained sections were subsequently examined and photographed using a light microscope (Axio Scope.A1, Zeiss, Germany).

### Electron Microscopy

Scanning Electron Microscopy (SEM). Fresh sperm samples from humans and mice were collected and resuspended in an electron microscopy fixative (Servicebio, G1102). The samples were fixed for 2 h at room temperature (RT) and subsequently incubated for an additional 8 h at 4°C. Sperm cells were then treated with 1% osmium tetroxide (OsO₄) in 0.1 m PBS (pH 7.4) for 1.5 h at room temperature. Following this, dehydration was performed using increasing concentrations of ethanol (30%, 50%, 70%, 90%, and 100%), with each concentration applied for 15 min. The samples were immersed in isoamyl acetate (Sinopharm, 10003128) for 20 min, and then dried using a critical point dryer (Quorum, K850). Finally, the samples were coated with gold particles and visualized using an S‐3400N scanning electron microscope (Hitachi, Tokyo, Japan). Transmission Electron Microscopy (TEM). In this study, mouse testes were dissected into small pieces and fixed overnight in 0.1 m cacodylate buffer (pH 7.4) containing 2.5% glutaraldehyde and 3% paraformaldehyde. Dehydration was achieved by progressively replacing the water in the samples with organic solvents such as ethanol or acetone. After dehydration, the samples were embedded in a resin, such as epoxy or acrylic, to preserve their structure during sectioning. This embedding process provides a solid matrix for cutting ultra‐thin slices of the samples. The embedded samples were then cut into ultra‐thin sections, typically between 50 and 100 nm thick, using an ultramicrotome. These ultrathin sections were counterstained with uranyl acetate and lead citrate before being examined with a JEM‐1400 transmission electron microscope (JEOL).

### Quantitative Real‐Time PCR (qRT‐PCR)

qRT‐PCR involves the extraction of total RNA from cells or tissues using commercially available RNA extraction kits or phenol‐chloroform (Invitrogen, 15596‐025), following the manufacturer's protocol. The quality and quantity of RNA should be assessed using spectrophotometry (e.g., by measuring the A260/280 ratio) or by determining the RNA integrity number (RIN) to ensure reliable results. RNA samples are then converted into complementary DNA (cDNA) through a reverse transcription reaction, which utilizes a reverse transcriptase enzyme and primers (either random hexamers or oligo‐dT) to synthesize cDNA from the RNA template. Relative mRNA expression levels are calculated using the 2^‐ΔΔCt^ method, with GAPDH serving as the internal control. Primer sequences are provided in Supplementary Table S5.

### Immunofluorescence Staining

Testes were freshly isolated and fixed in a 4% paraformaldehyde (PFA) solution (Sigma, P6148) for 4–6 h at 4°C. Sperm from the epididymis were fixed in 4% PFA and smeared onto slides. Following fixation, tissues were subjected to a sucrose gradient, embedded in O.C.T. compound (Sakura Finetek, 4583), and sectioned to a thickness of 5 µm. Chromosome spreading was performed to analyze the meiotic stages of spermatocytes, as previously described.^[^ ^30^ ^]^ Briefly, spermatocytes were fixed on glass slides using 1% paraformaldehyde. For immunofluorescence staining, cryosections underwent antigen retrieval by microwaving in 0.01 m sodium citrate buffer (pH 6.0) for 15 min. The sections were then permeabilized with 0.3% Triton X‐100 for 35 min, followed by blocking in 5% bovine serum albumin (BSA, AP0027, Amresco) in PBS for 1 h at room temperature. The chromosome spreading slides were blocked with antibody dilution buffer (ADB) for 30 min. After blocking, both cryosections and slides were incubated with primary and secondary antibodies, mounted with Hoechst 33342 to visualize cell nuclei and distinguish them from other structures, and imaged using a laser scanning confocal microscope (LSM900, Zeiss, Germany). Washing steps are essential to remove unbound primary and secondary antibodies, thereby reducing background staining. The samples were washed several times with PBS. Details of the primary and secondary antibodies used are provided in Supplementary Table S6.

### SKAP2 Protein Synthesis and Purification

The PEGX‐6P‐*Skap2* plasmid was constructed using the ClonExpress Ultra One Step Cloning Kit (C115, Vazyme). BL21(DE3) competent cells were transfected with the human or mouse SKAP2 cDNA sequence integrated into the PEGX‐6P plasmid, followed by a 12‐h culture period to facilitate protein expression. The transfected BL21(DE3) cells were harvested by ultracentrifugation at 18 000g for 20 min. After extracting protein, the supernatant was transferred to new 50 mL tubes. Subsequently, GST‐Flag beads and chromatography were employed to purify the SKAP2 protein.

### Isolation of Milk‐Derived Extracellular Vesicles (mEVs)

To generate the extracellular vesicles‐SKAP2 system, extracellular vesicles were initially isolated from raw milk through differential centrifugation, as previously described.^[^ ^49^, ^50^ ^]^ In brief, raw milk was centrifuged at 13 000g for 35 min to remove fat globules and cell debris. The upper fat layer and the bottom pellet were discarded, and the supernatant was collected. Centrifugation of the supernatant at 100 000g for 55 min was performed to remove large particles and microvesicles. Following centrifugation of the supernatant at 145 000g for 90 min, the extracellular vesicles pellet was retrieved, washed three times with PBS, and filtered through a 0.22 µm membrane.

### Preparation and Characterization of SKAP2‐Loaded mEVs

The engineered milk‐derived extracellular vesicles (mEVs) were loaded with SKAP2 via electroporation using the CUY21EDIT II (BEX, Japan) electroporation system. SKAP2 and extracellular vesicles were mixed in a 1:1 mass ratio in PBS, achieving a final extracellular vesicles concentration in the mixture was 0.1 mg mL^−1^. The blend was moved into 0.4‐cm cuvettes chilled on ice and subjected to electroporation for 10 cycles with a perforation voltage of 110 V, an opening time of 6 ms, an interval of 10 ms, a penetration voltage of 25 V, and a capacitance of 940 µF. After electroporation, the mixture was placed in a new tube and incubated at 37°C for 50 min to restore the integrity of the extracellular vesicles membrane. Transmission electron microscopy (TEM) and nanoparticle tracking analysis (NTA) were used to examine the morphology and size distribution of extracellular vesicles in each sample. The protein concentration was measured using the Pierce BCA Protein Assay Kit (Aspen, China) following the manufacturer's guidelines.

### In Vitro Fertilization

PMSG (5‐10 IU) was administered to the donor mice at 18:00 on Day 1, followed by an hCG injection (5–10 IU) administered 44–48 h later. HTF was equilibrated in a CO_2_ incubator at 37°C in the afternoon. After euthanizing the male mice, sperm was extracted from the epididymides and placed in HTF droplets, which were then overlaid with paraffin oil and incubated in the CO_2_ incubator for 1 h. Sperm‐egg co‐culture was maintained in the CO_2_ incubator for 4–6 h. The embryos were subsequently identified in the fertilization medium, carefully isolated, and washed multiple times with culture medium. Finally, the embryos were transferred to fresh medium for further culture.

### STA‐PUT Spermatogenic Cell Sorting

The mouse testis was carefully excised and rinsed in a Petri dish containing DMEM. The testis was then incubated in 5 mL of collagenase solution within a 15 mL centrifuge tube, placed in a 37°C water bath for 3–5 min, with gentle side‐to‐side shaking. Following the removal of the collagenase, 5 mL of pancreatic enzyme solution (containing DNase) was added, and digestion continued at 37°C for an additional 5–10 min. The reaction was terminated by the addition of 5 mL of DMEM with 10% BSA. The resulting suspension was filtered through a 70 µm filter and centrifuged at 500 g for 5 min. The cell pellet was then resuspended in a gradient separation solution, layered gently from the top, and incubated for 1.5–2 h. The final sample was collected for microscopic examination.

### DIA (Data Independent Acquisition) Quantitative Proteomics

Peptides were extracted from each sample and chromatographically separated using the Vanquish Neo UHPLC system (Thermo Scientific). The mobile phase comprised two buffers, Buffer A, which contained 0.1% formic acid in water, and Buffer b, consisting of 0.1% formic acid in acetonitrile (80% acetonitrile). The chromatographic column was equilibrated with 96% Buffer A. Following sample injection onto the Trap Column (PepMap Neo 5 µm C18, 300 µm × 5 mm, Thermo Scientific), peptide separation was conducted on the analytical column (µPAC Neo High Throughput Column, Thermo Scientific) using gradient elution. The gradient conditions were as follows, 0–0.1 min, 4% to 6% Buffer B, 0.1–1.1 min, 6% to 12% Buffer B, 1.1–4.3 min, 12% to 22.5% Buffer B, 4.3–6.1 min, 22.5% to 45% Buffer B, and 6.1–8 min, 99% Buffer B. After peptide separation, DIA mass spectrometry was performed using the Orbitrap Astral mass spectrometer (Thermo Scientific). The analysis lasted for 8 min, employing an electrospray voltage of 2.2 kV in positive ion mode, with a parent ion scan range of 380–980 m z^−1^. The resolution for the first stage of mass spectrometry was set to 240 000, with an AGC target of 500% and a maximum injection time (IT) of 3 ms. The resolution for the second stage of mass spectrometry was 80,000, also with an AGC target of 500% and a maximum IT of 3 ms. The RF‐lens was adjusted to 40%, with HCD (Higher‐energy Collisional Dissociation) as the MS2 activation type, an isolation window of 2 Th, and a normalized collision energy of 25%. The cycle time was 0.6 s. We extend our gratitude to Bioprofile for their contributions to proteomics.

### Cell Culture and Flow Cytometry Analysis

GC‐1, GC‐2, and TM3 cells were obtained from the Stem Cell Bank of the Chinese Academy of Sciences and cultured in the appropriate medium. All culture media were supplemented with 10% fetal bovine serum (FBS) and 1% penicillin‐streptomycin, and the cells were maintained at 37°C in a 5% CO_2_ incubator. To facilitate cell exposure to PTFE, PTFE microspheres were obtained from Jiangsu Zhichuan Technology Co., Ltd. PTFE was added into the culture medium to prepare the solution of 12.5 mg L^−1^ specific concentration. Early apoptosis, characterized by DNA strand breaks, was detected using the Annexin V‐FITC Apoptosis Detection Kit (MultiSciences, China) following the instructions provided in previous studies.^[^ ^51^, ^52^ ^]^ Briefly, cells were washed twice with cold PBS, gently trypsinized, and resuspended in 500 µL of 1 × binding buffer. The cells were then incubated with 5 µL of Annexin V‐FITC conjugate and 10 µL of propidium iodide (PI) solution at room temperature in the dark for 15 min. Following incubation, the cells were immediately analyzed using a flow cytometer.

### Seminiferous Tubule Microinjection

To investigate the potential signaling pathway of SKAP2 in PTFE‐induced reproductive damage, a short hairpin RNA (shRNA) targeting SKAP2 was designed using lentiviral vectors (Hanbio, China). Eight‐week‐old male mice were anesthetized, and their testes were exposed following standard surgical procedures. Lentiviral vectors containing the SKAP2‐targeting shRNA were introduced into the seminiferous tubules of the testes using glass micropipettes through the efferent ducts. Additionally, either extracellular vesicles‐control or extracellular vesicles‐SKAP2 was injected into the seminiferous tubules through the efferent ducts.

### Isolation and Identification of Leydig Cells

Mice were euthanized via cervical dislocation, and the testes were rinsed with PBS buffer. The tunica albuginea and surface blood vessels were meticulously removed using fine tweezers, ensuring the integrity of the seminiferous tubules. A combined enzymatic and mechanical method was employed, wherein a digestion solution containing 0.1% collagenase IV (1 mg mL^−1^) was added, and the mixture was incubated at 37°C with shaking at 100 rpm for 15 min. Following digestion, the tissue was gently separated with a pipette and tweezers to disaggregate the seminiferous tubules into a fluffy network. The stromal cells were isolated, and digestion was halted by adding an equal volume of culture medium. The suspension was allowed to stand for 2 min to facilitate cell settlement, after which it was filtered through a 200‐mesh filter, and the filtrate was collected. The suspension was centrifuged at 1000 rpm for 3–5 min, the supernatant was discarded, and the pellet was washed with PBS. After a second centrifugation at 1000 rpm for 3–5 min, the supernatant was again discarded, and the pellet was gently resuspended in 2–3 mL of leydig cell culture medium. The suspension was carefully layered onto a Percoll density gradient and centrifuged at 800 g for 30 min. Cells were collected from the 50%–60% layer, specifically at the upper boundary between 50% and 60%, and transferred to a new centrifuge tube. The pellet was washed with PBS and centrifuged at 1000 rpm for 3–5 min. This washing process was repeated with culture medium. After purification, leydig cells were resuspended in culture medium, diluted to a concentration of 5 × 10^5 cells mL^−1^ using serum‐containing medium, and inoculated (0.5 mL per well) into a 24‐well culture plate. The cells were then incubated in a CO_2_ incubator at 34°C with 100% humidity and 5% CO_2_. Purity Identification through 3β‐HSD Staining. Following 24 h of culture, the medium was removed and replaced with an adequate volume of staining solution. The cells were then incubated for a minimum of 2 h. Microscopic examination revealed that leydig cells displayed a deep blue coloration in the cytoplasm, thereby confirming their purity.

### Molecular Docking and In Vitro Verification

Molecular docking was performed according to the procedures described in a previous study.^[^ ^53^ ^]^ The initial protein structure was processed using AutoDock Tools 1.5.6, including hydrogen addition, charge assignment, docking atom type definition, and generation of a PDBQT file for docking. The PTFE molecule was retrieved using its CAS number to obtain the MOL structure, and its structure was optimized using the MMFF94 force field. The PTFE structure was then processed with AutoDock Tools 1.5.6 by adding hydrogens, calculating charges, assigning docking atom types, and generating a PDBQT file for docking. Docking was performed using AutoDock Vina 1.2.3 with a grid spacing of 0.375 Å, while other parameters were set to default values. The top 50 docking poses with the highest scores were obtained. Finally, the docking results and molecular interactions were visualized using PyMOL 2.5.0 and LigPlot 2.2.9 (scores below –7 kcal mol^−1^ typically reflect strong interactions). We appreciate phadcalc (www.phadcalc.com) for the molecular docking simulation. The 293T cell line carrying the *Skap2* point mutation was generated using the base editor hy(e)A3A‐BE4max, kindly provided by Prof. Dali Li (East China Normal University), following the protocol outlined in a previous publication.^[^ ^54^, ^55^ ^]^

### Statistical Analysis

Data are presented as the mean ± standard deviation (SD). Group differences were assessed using a two‐sided Student's *t*‐test in GraphPad Prism version 9.5.0. For datasets exhibiting skewed distributions, non‐parametric tests were utilized. Statistical significance was determined at ^*^ *p* < 0.05, ^**^ *p* < 0.01, and ^***^ *p* < 0.001.

## Author Contributions

S.G., S.Z., J.Z., and G.Z. contributed equally to this paper. S.G., C.Z., and H.H. conceived and designed the study. S.G., S.Z., J.Z., G.Z., J.C., R.L., K.S., S.L., and Y.W. carried out the majority of the bench work and performed data analysis. W.X. conducted the mouse IVF experiments and offered experimental guidance. Y.L. collected the human sperm samples and performed co‐incubation experiments. S.G., and S.Z. authored the manuscript. J.S., Y.Z., J.R., and H.H. provided academic consultation. C.Z. and H.H. contributed to the manuscript revision. All authors have read and approved the final version of the manuscript.

## Conflict of Interest

The authors declare no conflict of interest.

## Supporting information



Supporting Information

Supplemental Tables

## Data Availability

The data that support the findings of this study are available from the corresponding author upon reasonable request.

## References

[1] M. L. Eisenberg , S. C. Esteves , D. J. Lamb , J. M. Hotaling , A. Giwercman , K. Hwang , Y.‐S. Cheng , Nat. Rev. Dis. Primers 2023, 9, 49. 37709866 10.1038/s41572-023-00459-w

[2] Y. Liu , Q. Feng , L. Zou , C. Zhu , W. Xia , Andrology 2024. 10.1111/andr.13786 39435863

[3] Q. Zhao , Z. Fang , P. Wang , Z. Qian , Y. Yang , L. Ran , J. Zheng , Y. Tang , X. Cui , Y.‐Y. Li , Z. Zhang , H. Jiang , ACS Nano 2025, 19, 5589. 39869919 10.1021/acsnano.4c15112

[4] P. Zhu , S. Dong , P. Sun , A. P. Belgaumkar , Y. Sun , X. Cheng , Q. Zheng , T. Li , Cochrane Database Syst. Rev. 2023, 8, Cd012358. 37531575 10.1002/14651858.CD012358.pub2 PMC10400379

[5] A. K. Sarcon , O. A. Selim , B. L. Mullen , B. F. Mundell , S. L. Moran , K. R. Shen , J. Thorac. Cardiovasc. Surg. 2025, 1, 303. 10.1016/j.jtcvs.2024.05.026 38879120

[6] M. Cole , A. Gomiero , A. Jaén‐Gil , M. Haave , A. Lusher , Sci. Total Environ. 2024, 929, 172577. 38641111 10.1016/j.scitotenv.2024.172577

[7] C. Zhang , G. Zhang , K. Sun , J. Ren , J. Zhou , X. Liu , F. Lin , H. Yang , J. Cao , L. Nie , P. Zhang , L. Zhang , Z. Wang , H. Guo , X. Lin , S. Duan , J. Cao , H. Huang , EBioMedicine 2024, 108, 105369. 39342804 10.1016/j.ebiom.2024.105369 PMC11663775

[8] L. Tong , S. Zhang , Q. Liu , C. Huang , H. Hao , M. S. Tan , X. Yu , C. K. L. Lou , R. Huang , Z. Zhang , T. Liu , P. Gong , C. Han Ng , M. Muthiah , G. Pastorin , Sci. Adv. 2023, 15, ade5041. 10.1126/sciadv.ade5041 PMC10096581 37043568

[9] S. V. Krylova , D. Feng , Int. J. Mol. Sci. 2023, 24, 1337. 36674857 10.3390/ijms24021337 PMC9865891

[10] A. Javdani‐Mallak , I. Salahshoori , Sci. Total Environ. 2024, 925, 171774. 38508246 10.1016/j.scitotenv.2024.171774

[11] S. Sheller‐Miller , E. Radnaa , Y. Arita , D. Getahun , R. J. Jones , M. R. Peltier , R. Menon , Placenta 2020, 89, 42. 31675489 10.1016/j.placenta.2019.10.008 PMC7024050

[12] M. Abu‐Halima , N. Ludwig , M. Hart , P. Leidinger , C. Backes , A. Keller , M. Hammadeh , E. Meese , Fertil. Steril. 2016, 5, 1061. 10.1016/j.fertnstert.2016.06.030 27424049

[13] J. S. Gabrielsen , L. I. Lipshultz , Fertil. Steril. 2019, 111, 881. 30955844 10.1016/j.fertnstert.2019.02.021

[14] V. Murdica , E. Giacomini , A. Alteri , A. Bartolacci , G. C. Cermisoni , N. Zarovni , E. Papaleo , F. Montorsi , A. Salonia , P. Viganò , R. Vago , Fertil. Steril. 2019, 111, 897. 31029245 10.1016/j.fertnstert.2019.01.030

[15] V. Murdica , G. C. Cermisoni , N. Zarovni , A. Salonia , P. Viganò , R. Vago , Human Reprod. 2019, 8, 1416. 10.1093/humrep/dez114 31355853

[16] S.‐W. He , B.‐H. Xu , Y. Liu , Y.‐L. Wang , M.‐H. Chen , L. Xu , B.‐Q. Liao , R. Lui , F.‐P. Li , Y.‐H. Lin , X.‐P. Fu , B.‐B. Fu , Z.‐W. Hong , Y.‐X. Liu , Z.‐Q. Qi , H.‐L. Wang , Cell Cycle 2017, 23, 2272. 10.1080/15384101.2017.1380126 PMC5788478 28933599

[17] M. Boras , S. Volmering , A. Bokemeyer , J. Rossaint , H. Block , B. Bardel , V. Van Marck , B. Heitplatz , S. Kliche , A. Reinhold , C. Lowell , A. Zarbock , J. Exp. Med. 2017, 3, 851. 10.1084/jem.20160647 PMC5339670 28183734

[18] G. Martinez , D. Cappetta , M. Telesca , K. Urbanek , G. Castaldo , M. Dhellemmes , V. G. Mele , T. Chioccarelli , V. Porreca , A.‐L. Barbotin , A. Boursier , F. Guillou , C. Coutton , S. Brouillet , A. De Angelis , L. Berrino , R. Pierantoni , G. Cobellis , R. Chianese , F. Manfrevola , Int. J. Biol. Sci. 2023, 7, 2234. 10.7150/ijbs.77166 PMC10158014 37151878

[19] J.‐H. Chang , C.‐H. Chou , J.‐C. Wu , K.‐M. Liao , W.‐J. Luo , W.‐L. Hsu , X.‐R. Chen , S.‐L. Yu , S.‐H. Pan , P.‐C. Yang , K.‐Y. Su , Commun. Biol. 2023, 6, 389. 37037996 10.1038/s42003-023-04778-2 PMC10086033

[20] M. Finkelstein , B. Megnagi , D. Ickowicz , H. Breitbart , Dev. Biol. 2013, 381, 62. 23791551 10.1016/j.ydbio.2013.06.014

[21] W. Oosterheert , B. U. Klink , A. Belyy , S. Pospich , S. Raunser , Nature 2022, 7935, 374. 10.1038/s41586-022-05241-8 PMC9646518 36289337

[22] H. Tan , W. Wang , C. Zhou , Y. Wang , S. Zhang , P. Yang , R. Guo , W. Chen , J. Zhang , L. Ye , Y. Cui , T. Ni , K. Zheng , Nat. Commun. 2023, 14, 2499. 37120627 10.1038/s41467-023-38199-w PMC10294715

[23] X. Xiong , S. Feng , X. Ma , K. Liu , Y. Gui , B. Chen , X. Fan , F. Wang , X. Wang , S. Yuan , Adv. Sci. 2025, 12, 2412196. 10.1002/advs.202412196 PMC11967818 39921484

[24] L. Yin , N. Jiang , W. Xiong , S. Yang , J. Zhang , M. Xiong , K. Liu , Y. Zhang , X. Xiong , Y. Gui , H. Gao , T. Li , Y. Li , X. Wang , Y. Zhang , Adv. Sci. 2024, 12, 2406332. 10.1002/advs.202406332 PMC11744674 39607422

[25] M. Liu , L. Wang , Y. Li , E. Zhi , G. Shen , X. Jiang , D. Li , X. Zhao , T. Ruan , C. Jiang , X. Wang , X. Zhang , Y. Zheng , B. Wu , N. Ou , G. Zhao , S. Dai , R. Zhou , L. Yang , Y. Yang , H. Liu , Y. Shen , Adv. Sci. 2024, 33, 2402412. 10.1002/advs.202402412 PMC11434121 38958533

[26] J.‐Y. Kang , Z. Wen , D. Pan , Y. Zhang , Q. Li , A. Zhong , X. Yu , Y.‐C. Wu , Y. Chen , X. Zhang , P.‐C. Kou , J. Geng , Y.‐Y. Wang , M.‐M. Hua , R. Zong , B. Li , H.‐J. Shi , D. Li , X.‐D. Fu , J. Li , D. L. Nelson , X. Guo , Y. Zhou , L.‐T. Gou , Y. Huang , M.‐F. Liu , Science 2022, 6607, abj6647. 10.1126/science.abj6647 35951695

[27] Y. Fan , C. Huang , J. Chen , Y. Chen , Y. Wang , Z. Yan , W. Yu , H. Wu , Y. Yang , L. Nie , S. Huang , F. Wang , H. Wang , Y. Hua , Q. Lyu , Y. Kuang , M. Lei , Sci. Bull. 2022, 20, 2112. 10.1016/j.scib.2022.09.026 36546111

[28] Y. Ma , Q. W. Ma , Y. Sun , X. F. Chen , Human Reprod. 2023, 3, 334. 10.1093/humrep/dead015 36728671

[29] Y. Shu , L. Zhang , J. He , L. Tang , Y. Wu , P. Hong , H. Wu , L. Chen , Chem. Res. Toxicol. 2025, 3, 478. 10.1021/acs.chemrestox.4c00498 39983089

[30] M. Lei , Z. Zhu , C. Wei , H. Xie , R. Guo , Y. Zhao , K. Wang , M. Wang , W. Chen , X. Xu , X. Zeng , Y. Xu , W. Zhang , Y. Chu , Y. Sun , Q. Yang , Adv. Sci. 2024, 12, 2410353. 10.1002/advs.202410353 PMC11744561 39574356

[31] G. Pujol , L. Marín‐Gual , L. González‐Rodelas , L. Álvarez‐González , F. Chauvigné , J. Cerdà , M. Teles , N. Roher , A. Ruiz‐Herrera , J. Hazard. Mater. 2025, 481, 136529. 39556913 10.1016/j.jhazmat.2024.136529

[32] M. W. Skinner , C. J. Simington , P. López‐Jiménez , K. A. Baran , J. Xu , Y. Dayani , M. V. Pryzhkova , J. Page , R. Gómez , A. J. Holland , P. W. Jordan , EMBO Rep. 2024, 25, 3373. 38943004 10.1038/s44319-024-00187-6 PMC11316026

[33] X. Tan , C. Zheng , Y. Zhuang , P. Jin , F. Wang , Nat. Commun. 2023, 14, 1636. 36964127 10.1038/s41467-023-37252-y PMC10039029

[34] S. Gan , S. Zhou , J. Ma , M. Xiong , W. Xiong , X. Fan , K. Liu , Y. Gui , B. Chen , B. Zhang , X. Wang , F. Wang , Z. Li , W. Yan , M. Ma , S. Yuan , EMBO Rep. 2024, 4, 2045 10.1038/s44319-024-00112-x PMC11015022 38454159

[35] M. Blanco , L. El Khattabi , C. Gobé , M. Crespo , M. Coulée , A. de la Iglesia , C. Ialy‐Radio , C. Lapoujade , M. Givelet , M. Delessard , I. Seller‐Corona , K. Yamaguchi , N. Vernet , F. Van Leeuwen , A. Lermine , Y. Okada , R. Daveau , R. Oliva , P. Fouchet , A. Ziyyat , D. Pflieger , J. Cocquet , EMBO Rep. 2023, 6, 56316. 10.15252/embr.202256316 PMC10240200 37099396

[36] C. Ma , J. Huang , Y. Jiang , L. Liu , N. Wang , S. Huang , H. Li , X. Zhang , S. Wen , B. Wang , S. Yang , EMBO Mol. Med. 2024, 16, 361. 38177538 10.1038/s44321-023-00016-8 PMC10897472

[37] X. Z. Zhang , L. L. Wei , H. J. Jin , X. H. Zhang , S. R. Chen , Cell Rep. 2022, 1, 111049. 10.1016/j.celrep.2022.111049 35793634

[38] L. M. Doyle , M. Z. Wang , Cells 2019, 8, 727.31311206

[39] J. Ai , S. Zhang , M. Dai , P. Jiang , J. Huang , H. Xiao , Y. Lin , X. Tang , W. Tong , J. He , Q. Mao , Y. Wang , Z. Ye , T. Wang , R. Chai , Adv. Sci. 2025, 02627. 10.1002/advs.202502627 PMC12376537 40411396

[40] C. Y. Li , S. P. Liu , X. F. Dai , D. F. Lan , T. Song , X. Y. Wang , Q. H. Kong , J. Tan , J. D. Zhang , Asian J. Androl. 2023, 5, 547. 10.4103/aja2022126 PMC10521952 37040218

[41] A. Kowalczyk , M. Wrzecińska , E. Czerniawska‐Piątkowska , R. Kupczyński , Biomed. Pharmacother. 2022, 148, 112752. 35220028 10.1016/j.biopha.2022.112752

[42] W. C. Chang , S. H. Li , P. S. Tsai , in Advances in Anatomy, Embryology, and Cell Biology, Springer, Berlin 2024.

[43] R. Deng , Z. Wu , C. He , C. Lu , D. He , X. Li , Z. Duan , H. Zhao , PeerJ. 2024, 12, 16875. 10.7717/peerj.16875 PMC11056104 38680889

[44] W. Ali , K. Deng , Y. Bian , Z. Liu , H. Zou , Biomed. Pharmacother. 2023, 164, 114889. 37209627 10.1016/j.biopha.2023.114889

[45] T. Reyes‐Miguel , A. L. Roa‐Espitia , R. Baltiérrez‐Hoyos , E. O. Hernández‐González , Reproduction 2020, 3, 393. 10.1530/REP-19-0577 32567555

[46] L. Zhou , H. Liu , S. Liu , X. Yang , Y. Dong , Y. Pan , Z. Xiao , B. Zheng , Y. Sun , P. Huang , X. Zhang , J. Hu , R. Sun , S. Feng , Y. Zhu , M. Liu , M. Gui , J. Wu , Cell 2023, 186, 2897. 37295417 10.1016/j.cell.2023.05.009

[47] W.‐H. J. Ho , M. B. Marinova , D. R. Listijono , M. J. Bertoldo , D. Richani , L.‐J. Kim , A. Brown , A. H. Riepsamen , S. Cabot , E. R. Frost , S. Bustamante , L. Zhong , K. Selesniemi , D. Wong , R. Madawala , M. Marchante , D. M. Goss , C. Li , T. Araki , D. J. Livingston , N. Turner , D. A. Sinclair , K. A. Walters , H. A. Homer , R. B. Gilchrist , L. E. Wu , EMBO Mol. Med. 2024, 16, 2583. 39169162 10.1038/s44321-024-00119-w PMC11473878

[48] S. Lee , K. K. Kang , S. E. Sung , J. H. Choi , M. Sung , K. Y. Seong , J. Lee , S. Kang , S. Y. Yang , S. Lee , K. R. Lee , M. S. Seo , K. Kim , Polymers 2022, 14, 402.35160391

[49] H. Bao , Y. Tian , H. Wang , T. Ye , S. Wang , J. Zhao , Y. Qiu , J. Li , C. Pan , G. Ma , W. Wei , Y. Tao , Nat. Biomed. Eng. 2024, 11, 1436. 10.1038/s41551-023-01112-3 37872369

[50] K. Wang , X. Zhao , S. Yang , X. Qi , G. Zang , C. Li , A. Li , B. Chen , Crit. Rev. Food Sci. Nutr. 2024, 65, 2388. 10.1080/10408398.2024.2338831 38595109

[51] S. Gan , M. Yang , L. Fan , L. Xie , Y. Xu , B. Wang , T. Xu , L. Yu , J. Ma , W. Chen , Chem. Res. Toxicol. 2020, 5, 1256. 10.1021/acs.chemrestox.0c00018 32223187

[52] E. W. F. Cordeiro , E. L. Marzola , R. S. Maekawa , M. R. D. Santos , L. G. Assunção , M. P. Massafera , J. D. Oliveira , T. G. C. Batista , M. C. O. P. D. Sales , S. S. Maria‐Engler , P. Di Mascio , M. H. G. D. Medeiros , G. E. Ronsein , A. P. D. M. Loureiro , Chem. Res. Toxicol. 2024, 8, 1246. 10.1021/acs.chemrestox.3c00312 PMC11337214 38990804

[53] L. Zhou , G. Lian , T. Zhou , Z. Cai , S. Yang , W. Li , L. Cheng , Y. Ye , M. He , J. Lu , Q. Deng , B. Huang , X. Zhou , D. Lu , F. Zhi , J. Cui , Nat. Cancer 2025, 6, 768. 40108413 10.1038/s43018-025-00937-y

[54] X. Zhang , L. Chen , B. Zhu , L. Wang , C. Chen , M. Hong , Y. Huang , H. Li , H. Han , B. Cai , W. Yu , S. Yin , L. Yang , Z. Yang , M. Liu , Y. Zhang , Z. Mao , Y. Wu , M. Liu , D. Li , Nat. Cell Biol. 2020, 22, 740 32393889 10.1038/s41556-020-0518-8

[55] S. Dupuis , M. S. Girault , M. Le Beulze , C. Ialy‐Radio , L. Bermúdez‐Guzmán , A. Ziyyat , S. Barbaux , Cell. Mol. Biol. Lett. 2024, 29, 10.1186/s11658-024-00587-5 PMC11094962 38750428

