# Supplementary material for: Therapeutic Repair of Sperm Quality Decline Caused by Polytetrafluoroethylene
Source: Adv Sci (Weinh). 2025 Jul 25;12(38):e05148. doi: 10.1002/advs.202505148 (PMC12520549; doi:10.1002/advs.202505148)
Supplement: Supplementary file 1 — Supporting Information [file ADVS-12-e05148-s002.pdf]

ADVANCED SCIENCE
Open Access

Supporting Information

for *Adv. Sci.*, DOI 10.1002/advs.202505148

Therapeutic Repair of Sperm Quality Decline Caused by Polytetrafluoroethylene

Shiming Gan, Shumin Zhou, Jiaming Zhou, Guanghui Zhang, Jingshou Chen, Rui Liu, Kuan Sun, Sisi Li, Wenjing Xiong, Yujiao Wen, Jianzhong Sheng, Yu Zhang, Jingchao Ren*, Youjiang Li*, Hefeng Huang* and Chen Zhang**

Supporting Information

Therapeutic Repair of Sperm Quality Decline Caused by Polytetrafluoroethylene

Shiming Gan, Shumin Zhou, Jiaming Zhou, Guanghui Zhang, Jingshou Chen, Rui Liu, Kuan Sun, Sisi Li, Wenjing Xiong, Yujiao Wen, Jianzhong Sheng, Yu Zhang, Jingchao Ren,* Youjiang Li,* Hefeng Huang,* Chen Zhang**

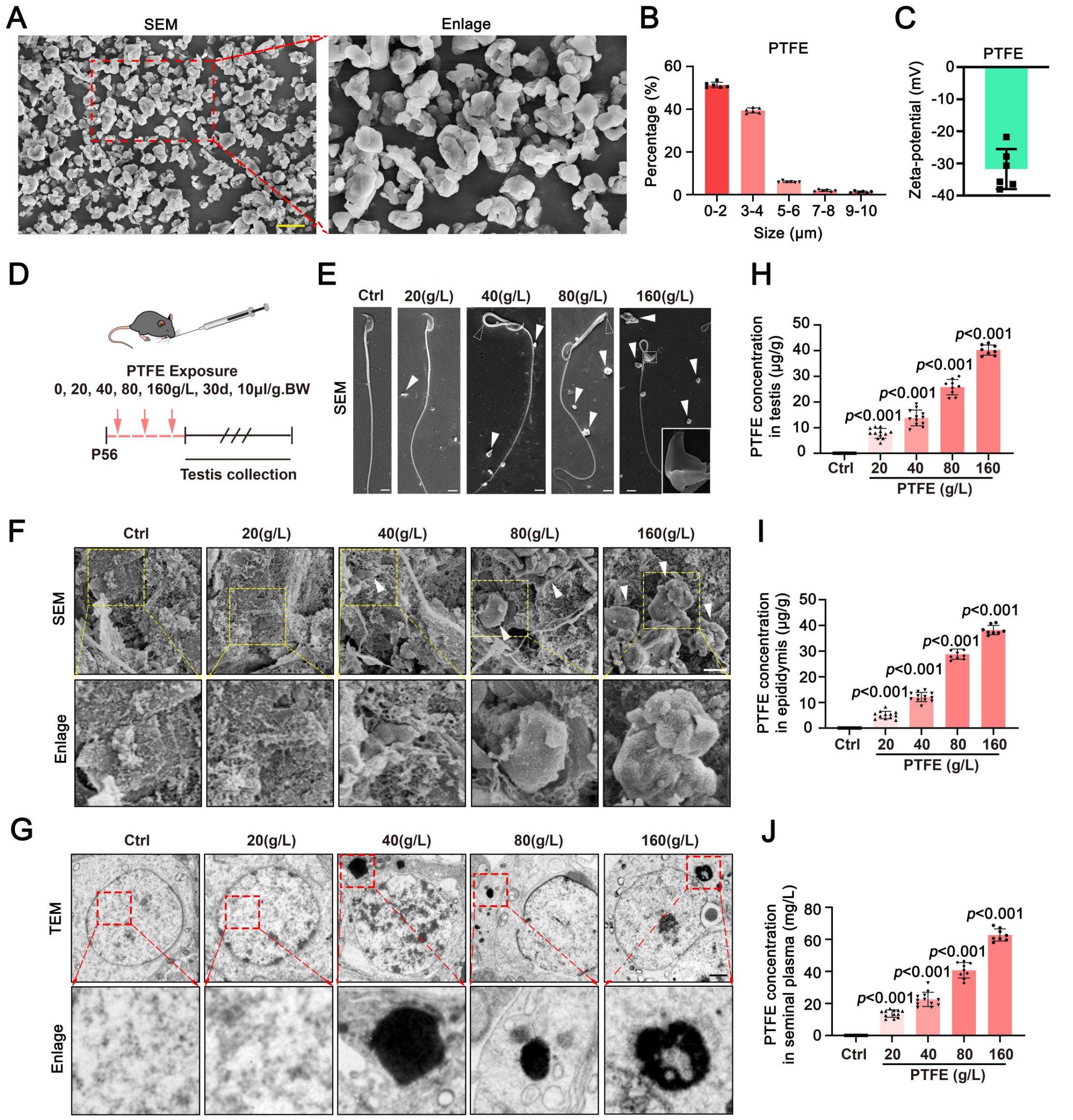

Figure S1. Characteristics and exposure of PTFE.

A,B) Representative scanning electron microscopy (SEM) images and measured diameters of PTFE particles are presented, with scale bars set at 5 μm. C) The ζ-potentials of PTFE were found to be negative, averaging between -38.06 mV and -21.77 mV. D) A schematic diagram illustrates the experimental project: 8-week-old wild-type male mice were administered varying doses of PTFE over a 30-day period, with dosages adjusted according to body weight. E) SEM ultrastructural analysis of epididymal sperm from control and PTFE-exposed mice is shown. Insets provide higher magnification images of sperm heads, with white arrowheads indicating PTFE particles and white dotted hollows highlighting malformed sperm heads and flagella. Scale bars are 5 μm. F) SEM ultrastructural analysis of testicular tissue from control

(left) and PTFE-exposed (right) mice is illustrated, with white arrowheads indicating PTFE particles. Scale bar is 1 μm. G) Transmission electron microscopy (TEM) ultrastructural analysis of seminiferous tubules from control (left) and PTFE-exposed (right) mice is depicted, with red dashed boxes indicating PTFE particles. Scale bars are 1 μm. H-J) Quantification of PTFE concentrations in the testis (H), epididymis (I), and seminal plasma (J) is presented, with data expressed as mean ± SD. p-values were determined using a two-sided Student's t-test compared with control.

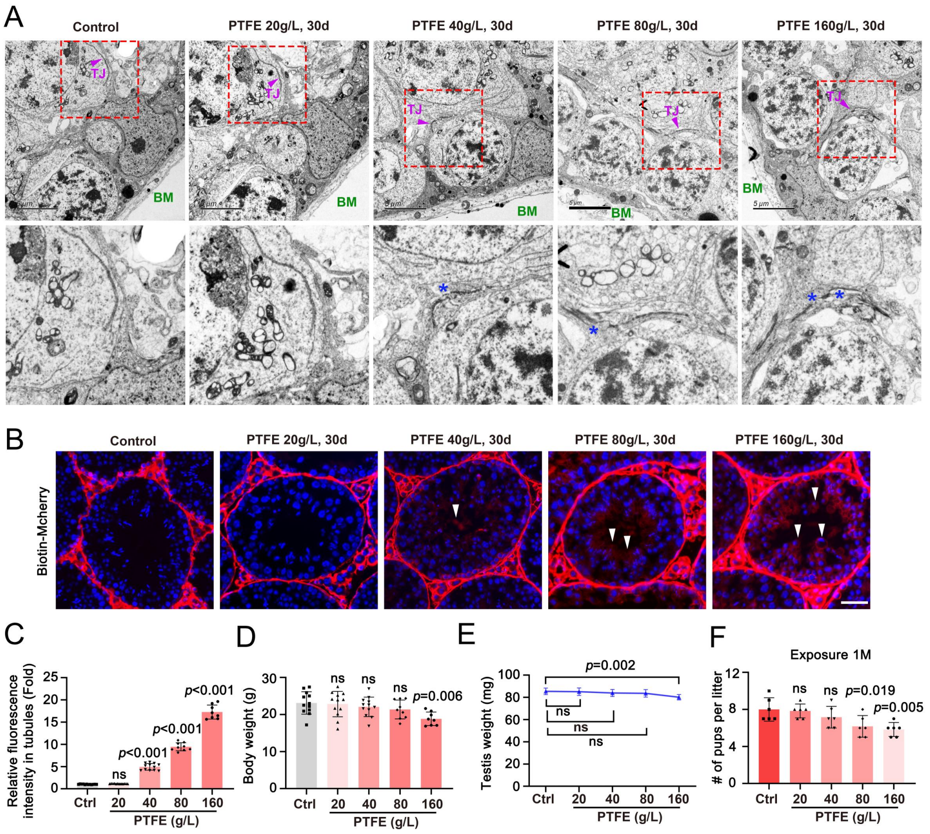

Figure S2. Exposure to PTFE results in damage to the blood-testis barrier (BTB) structure and spermatogenesis.

A) Ultrastructural damages of the BTB following PTFE exposure were assessed in control and PTFE-exposed mice using electron microscopy. The red dashed boxes in the upper panels indicate areas of higher magnification, while the lower panels correspond to these magnified regions. Purple arrowheads highlight the typical structure of tight junctions (TJs), and blue asterisks denote disruption of the BTB. Abbreviations: TJ, tight junction, BM, basal membrane. B) Immunofluorescence staining was conducted to evaluate BTB integrity in control and PTFE-exposed mice

through biotin tracer experiments. Biotin was injected beneath the testicular capsules, and frozen sections were stained with Streptavidin-Alexa Fluor 594 (red) and DAPI (blue) to visualize biotin localization. White arrowheads indicate biotin within the tubules. Scale bars=50 μm. C) Quantification of biotin fluorescence intensity in the testicular tubules from control and PTFE-exposed mice is presented as mean ± SD. NS indicates no significant difference. *p*-values were calculated using a two-sided Student's *t*-test compared with control. D) Quantification of body weight in control and PTFE-exposed mice. Data are expressed as mean ± SD. NS indicates no significant difference. *p*-values were determined using a two-sided Student’s *t*-test compared with control. E) Quantification of testis weight in control and PTFE-exposed mice. Data are expressed as mean ± SD. NS indicates no significant difference. *p*-values were determined using a two-sided Student’s *t*-test compared with control. F) Fertility test results for control and PTFE-exposed mice after one month exposure of PTFE. Data are expressed as mean ± SD. NS indicates no significant difference. *p*-values were determined using a two-sided Student’s *t*-test compared with control.

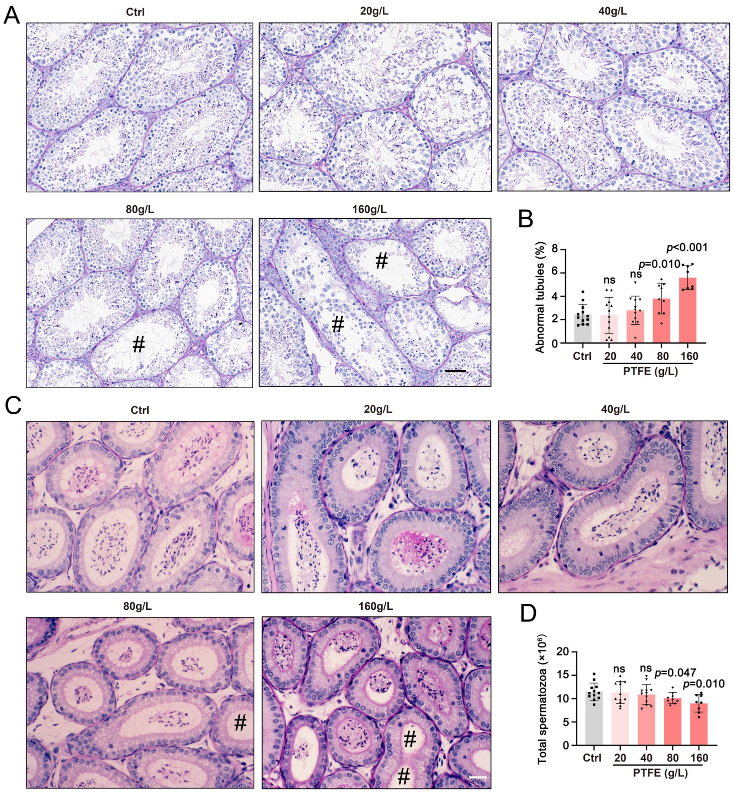

Figure S3. Impact of PTFE exposure on testicular tubule development and spermatogenesis.

A) Representative periodic acid-Schiff (PAS) staining images of seminiferous tubules from control and PTFE-exposed mice. Scale bars=50 μm. Tubules marked with # indicate abnormalities. B) Histogram illustrating the proportion of abnormal tubules in the testes of control versus PTFE-exposed mice. Data are presented as mean ± SD. NS=not significant. *p* values were determined using a two-sided Student's *t*-test compared with control. C) Histological analysis of epididymides caput from control and PTFE-exposed mice. Tubules marked with # indicate abnormalities. Scale bars=50 μm. D) Histogram displaying sperm counts from control and PTFE-exposed mice. Data are presented as mean ± SD. NS=not significant. *p* values were determined using a two-sided Student's *t*-test compared with control.

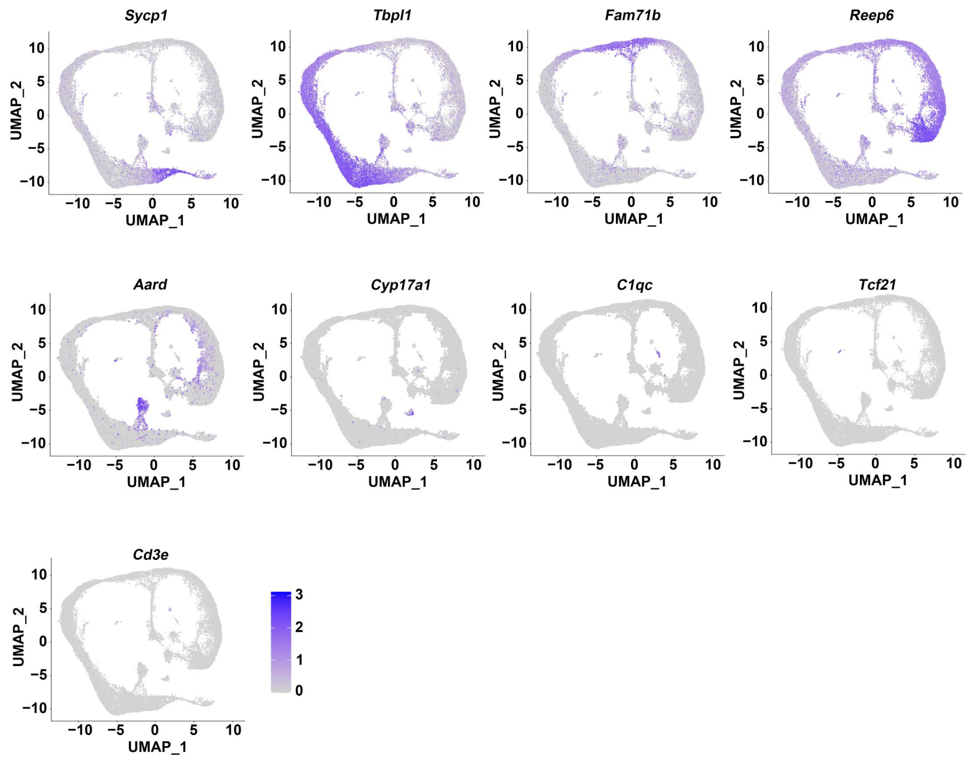

Figure S4. Representative gene expression patterns in cells, with each gene's expression scale presented independently.

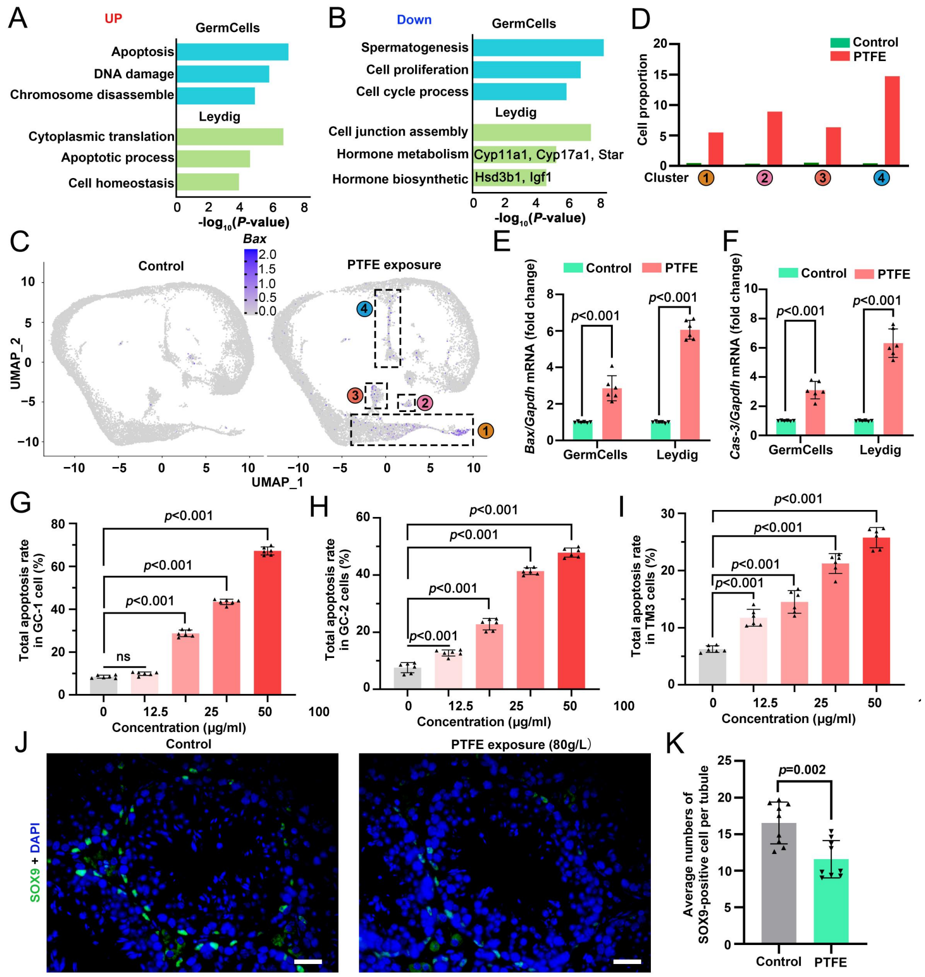

Figure S5. The apoptosis process induced by PTFE exposure.

A,B) Gene Ontology (GO) term analysis reveals upregulated (A) and downregulated (B) genes in germ cells and leydig cells from control and 80 g/L PTFE-treated mice. C) UMAP plots depict mRNA levels of the key apoptosis gene, *Bax*. D) A bar graph illustrates the proportion of apoptotic cells across the four clusters in control and 80 g/L PTFE-treated mice. E,F) qRT-PCR analysis of mRNA expression levels of *Bax* (E) and *Caspase-3* (F) in germ cells and leydig cells from control and 80 g/L PTFE-treated mice is shown. Data are presented as mean ± SD, with *p*-values determined by a two-sided Student's *t*-test. G-I) Total apoptosis rate in GC-1 cells (G), GC-2 cells (H), and TM3 cells (I) after exposure to different concentrations of PTFE (0, 12.5, 25, 50, 100 μg/ml). Data are presented as mean ± SD. *p* values were calculated using a two-sided Student's *t*-test compared with control. J) Representative immunofluorescence images displaying SOX9 (green) staining in the testis of control and PTFE-exposed mice. Scale bars=50 μm. K) Quantification of SOX9-positive

sertoli cell from panel (J). Data are presented as mean ± SD. Statistical significance was determined using a two-sided Student's *t*-test.

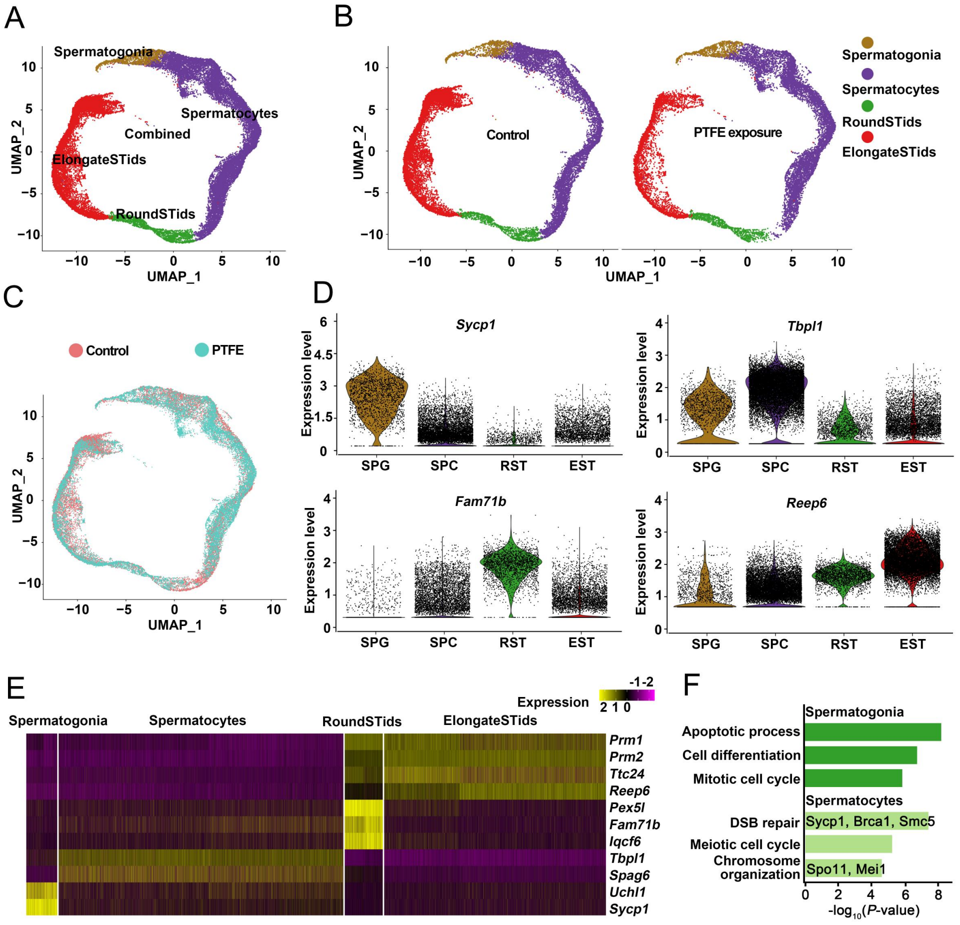

Figure S6. Single-cell RNA sequencing (scRNA-seq) delineates germ cell clusters. A) UMAP representation of single-cell RNA sequencing profiles from germ cells in both control and PTFE-exposed mouse testis. Each dot represents an individual cell, color-coded according to its cluster identity, as indicated in the figure. B) UMAP and clustering analysis of single-cell transcriptomes from control and PTFE-exposed mouse germ cells, with UMAP dimensional reduction results presented separately for each group. C) UMAP plot illustrating the clustering analysis of combined single-cell transcriptomic data from control and PTFE-exposed mouse germ cells, with clusters color-coded and labeled accordingly. D) Violin plots displaying the mean expression levels of selected marker genes for spermatogonia (SPG), meiotic spermatocytes (SPC), post-meiotic round spermatids (RST), and elongate spermatids (EST) across clusters. E) Heatmap depicting the expression of identity genes for spermatogonia,

spermatocytes, round spermatids, and elongate spermatids in individual germ cells. F) Gene Ontology (GO) enrichment analysis of differentially expressed genes in spermatogonia and spermatocytes.

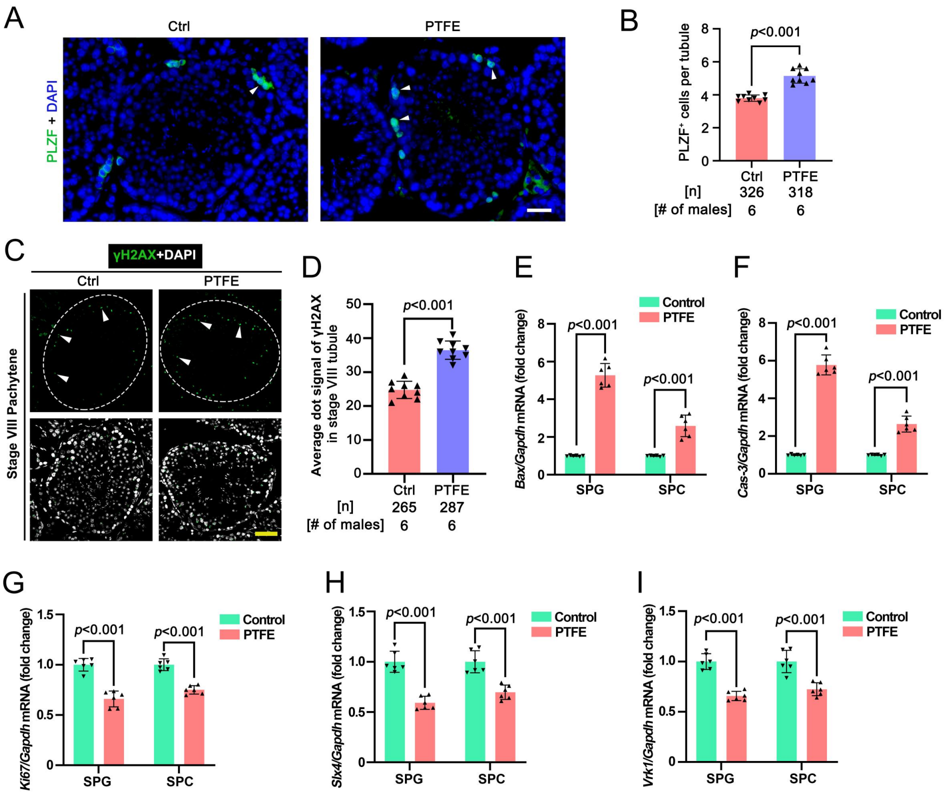

Figure S7. Aberrant progression of spermatogonia and spermatocytes exposed to PTFE.

A) Representative immunofluorescence images displaying PLZF (green) staining in the testis of control and PTFE-exposed mice. Arrowheads indicate PLZF-positive cells. Scale bars=50 μm. B) Quantification of PLZF-positive spermatogonia from panel (A). Data are presented as mean ± SD. Statistical significance was determined using a two-sided Student's *t*-test. C) Representative immunofluorescence images showing γH2AX (green) staining in the testis of control and PTFE-exposed mice. Arrowheads indicate γH2AX-positive cells. Scale bars=50 μm. D) Quantification of γH2AX-positive spermatocytes from panel (C). Data are presented as mean ± SD. Statistical significance was determined using a two-sided Student's *t*-test. E,F) qRT-PCR analysis of mRNA expression levels of *Bax* (E) and *Caspase-3* (F) in control and 80 g/L PTFE-exposed mouse spermatogonia (SPG) and spermatocytes

(SPC). Data are presented as mean ± SD. Statistical significance was determined using a two-sided Student's *t*-test. G-I) qRT-PCR analysis of mRNA expression levels of *Ki67* (G), *Slx4* (H), and *Vrk1* (I) in control and 80 g/L PTFE-treated mouse spermatogonia (SPG) and spermatocytes (SPC). Data are presented as mean ± SD. Statistical significance was determined using a two-sided Student's *t*-test.

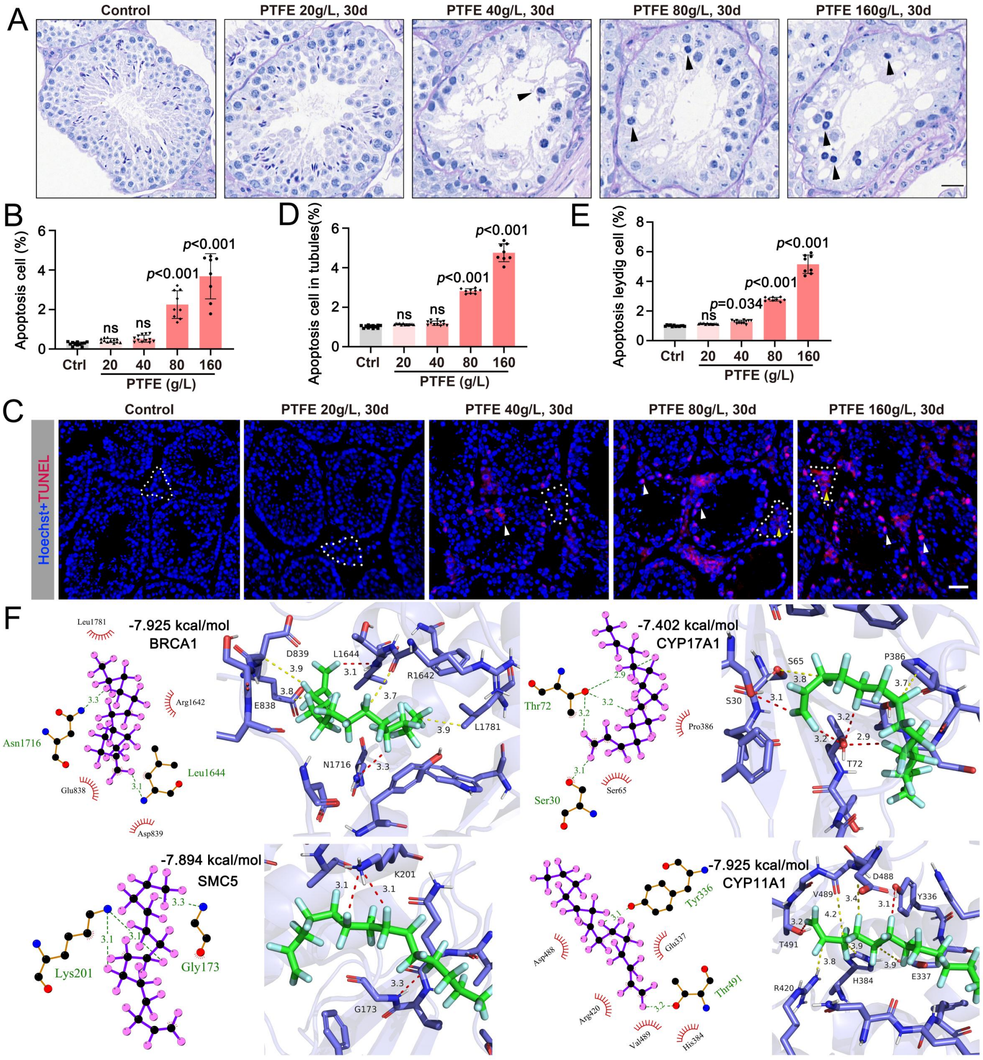

Figure S8. The apoptosis in mouse testis following PTFE exposure.

A) Representative images of periodic acid-Schiff (PAS) staining in the seminiferous tubules of control and PTFE-exposed mouse testis are presented. Black arrowheads indicate apoptotic germ cells, with scale bars measuring 50 μm. B) Quantification of apoptotic cells in control and PTFE-exposed mouse testis is depicted, as shown in panel (A). Data are presented as mean ± SD. *p* values were calculated using a two-sided Student's *t*-test compared with control. C) The TUNEL assay is employed

to detect apoptosis in control and PTFE-exposed mouse testis. Dotted lines delineate leydig cells, while white arrowheads highlight apoptotic cells within the tubules, and yellow arrowheads indicate apoptotic leydig cells. Scale bars are 50 μm. D,E) Quantification of apoptotic cells within the tubules (D) and leydig cells (E) is presented for both control and PTFE-exposed mouse testis, corresponding to panel (C). Data are presented as mean ± SD. *p* values were calculated using a two-sided Student's *t*-test compared with control. F) Docking model of PTFE molecules with the proteins. In the 2D interaction diagram (left), the Van der Waals and hydrophobic interactions are indicated by eyelash-like symbols, and hydrogen bonds are represented by green dotted lines. The 3D structural diagram (right) shows the hydrogen bonds as red dotted lines, and van der Waals interactions as yellow dotted lines.

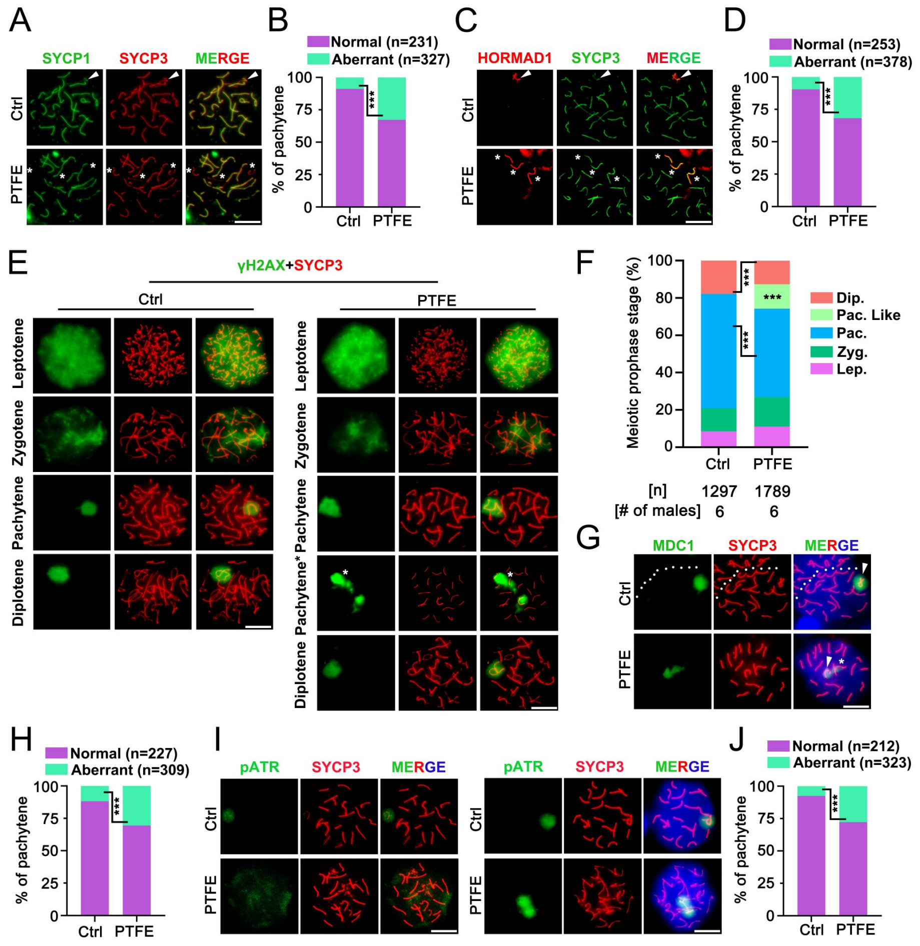

Figure S9. Defective synapsis and DNA damage response in PTFE-exposed pachytene spermatocytes.

A) Chromosome spreads from control and PTFE-exposed pachytene spermatocytes were immunostained with SYCP1 (green) and SYCP3 (red) antibodies. SYCP1 and SYCP3 label the central and lateral elements of the synaptonemal complex, respectively. White arrowheads indicate the pairing of sex chromosomes in the pseudoautosomal region (PAR), while asterisks denote the unsynapsed autosomes, characterized by the loss of SYCP1 signals on chromosomes. Scale bar: 10 μm. **B)** Histogram illustrating the quantitative analysis of spermatocytes from control and 80 g/L PTFE-exposed mice. Data are presented as mean ± SD. ***$P<0.001$, Student's *t*-test (two-sided). **C)** Co-immunostaining of HORMAD1 (red) and SYCP3 (green) in control and PTFE-exposed pachytene spermatocytes. White arrowheads indicate the sex chromosomes, while asterisks highlight unsynapsed chromosomes. Scale bar: 10 μm. **D)** Histogram depicting the quantitative analysis of spermatocytes from control and 80 g/L PTFE-exposed mice. Data are presented as mean ± SD. ***$P<0.001$, Student's *t*-test (two-sided). **E)** Step-by-step co-immunostaining of γH2AX (green) and SYCP3 (red) in control and PTFE-exposed spermatocytes. Asterisks indicate the mislocalization of γH2AX on unsynapsed autosomes. Scale bar: 10 μm. **F)** Histogram showing the percentage of spermatocytes from control and 80 g/L PTFE-exposed mice. Data are presented as mean ± SD. ***$P<0.001$, Student's *t*-test (two-sided). **G)** Co-immunostaining of MDC1 (green) and SYCP3 (red) was performed on control and PTFE-exposed pachytene spermatocytes. White arrowheads indicate the sex chromosomes located in the XY body, while asterisks denote ectopic MDC1 localization on unsynapsed autosomes. Scale bar: 10 μm. **H)** A histogram illustrates the quantitative analysis of spermatocytes from control and 80 g/L PTFE-exposed mice. Data are presented as mean ± SD, with statistical significance indicated as ***$P<0.001$, determined by Student's *t*-test (two-sided). **I)** Immunostaining of chromosome spreads from pachytene spermatocytes in control and 80 g/L PTFE-exposed testis was conducted using antibodies against SYCP3 (red) and P-ATR (green). Scale bar: 10 μm. **J)** Histogram presents the quantitative analysis of spermatocytes from control and 80 g/L PTFE-exposed mice. Data are expressed as mean ± SD, with statistical significance marked as ***$P<0.001$, based on Student's

t-test (two-sided).

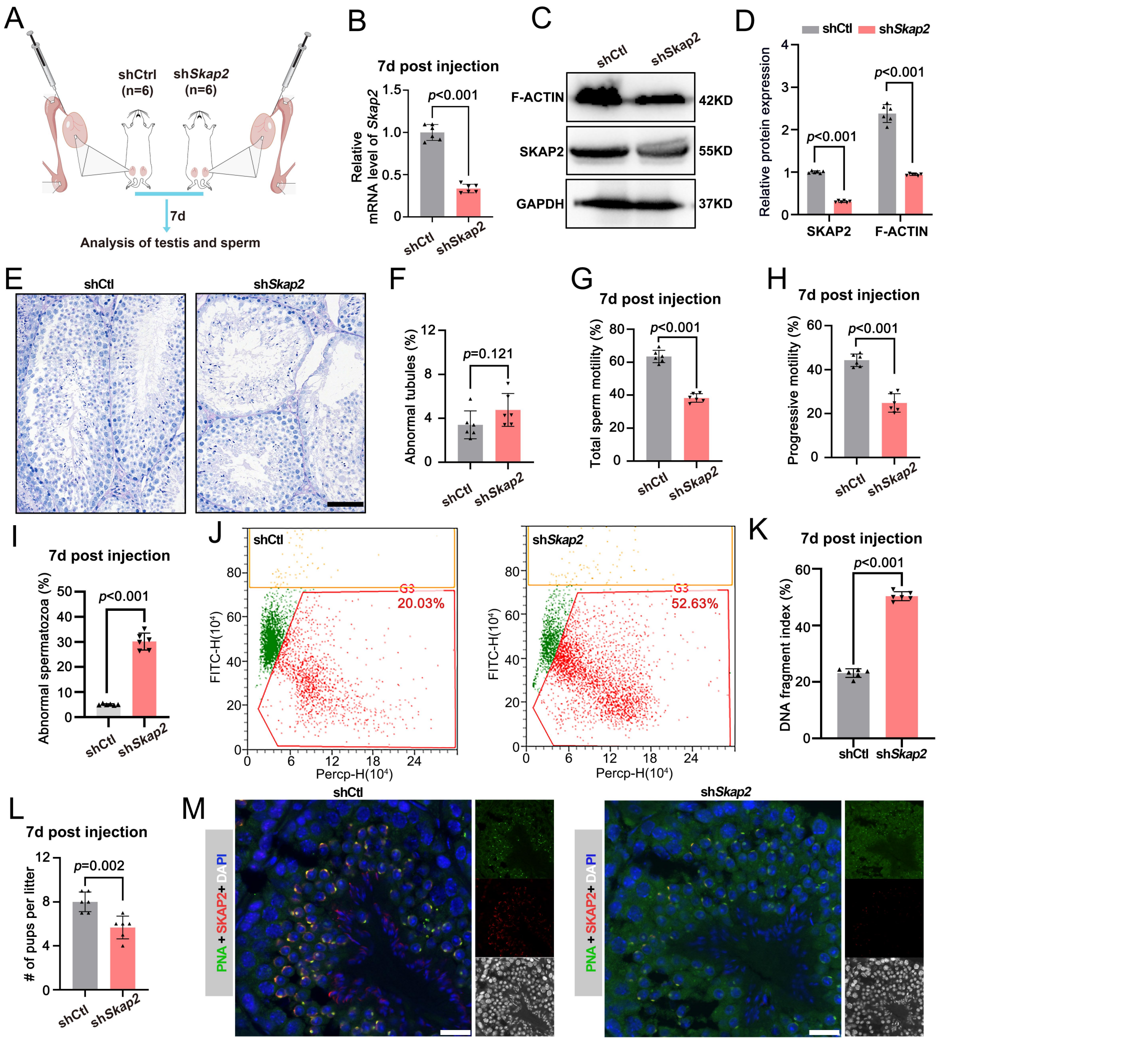

Figure S10. SKAP2 knockdown impairs spermiogenesis and reduces fertility.

A) A diagram illustrates the injection of either control-shRNA or Skap2-shRNA lentivirus into mouse testis via the efferent ducts. Seven days post-injection, testis and sperm were collected and analyzed. B) RT-qPCR analysis indicates a significant decrease in Skap2 mRNA expression following 7 days of shSkap2 injection compared to the shCtl group. Data are presented as mean ± SD, with *p*-values calculated using a two-sided Student's *t*-test. C) Representative western blot showing that SKAP2 and F-ACTIN protein was decreased in shSkap2 testis compared to the shCtl group. GAPDH served as a loading control. D) Quantification of SKAP2 and F-ACTIN protein level in shCtrl and shSkap2 testis. Data are presented as mean ± SD. *p* values (Student *t*-test, two-sided). E) Representative images of the periodic acid-Schiff (PAS) staining of shCtrl and shSkap2 mouse seminiferous tubules are shown. Scale bars =

50 μm. F) Quantifying the percentage of abnormal seminiferous tubules from shCtrl and shSkap2 mice. Data are presented as mean ± SD. *p* values (Student *t*-test, two-sided). G,H) CASA analysis of the total sperm motility (G) and progressive motility (H) in shCtrl and shSkap2 mouse epididymis spermatozoa. Data are presented as mean ± SD. *p* values (Student *t*-test, two-sided). I) Quantification of the deformed spermatozoa in shCtrl and shSkap2. Data are presented as mean ± SD. *p* values (Student *t*-test, two-sided). J) Flow cytometry analysis of sperm DNA fragmentation ratios in the shCtl and shSkap2 groups. K) Quantification of the DNA fragmentation rate in shCtrl and shSkap2 mouse epididymis spermatozoa. Data are presented as mean ± SD. *p* values (Student *t*-test, two-sided). L) Litter size analysis for the shCtl and shSkap2 groups, with data presented as mean ± SD and *p*-values calculated using a two-sided Student's *t*-test. M) SKAP2 (red) staining with PNA (green) in testis after the injection of shCtl or shSkap2 seven days. Scale bar = 25 μm.

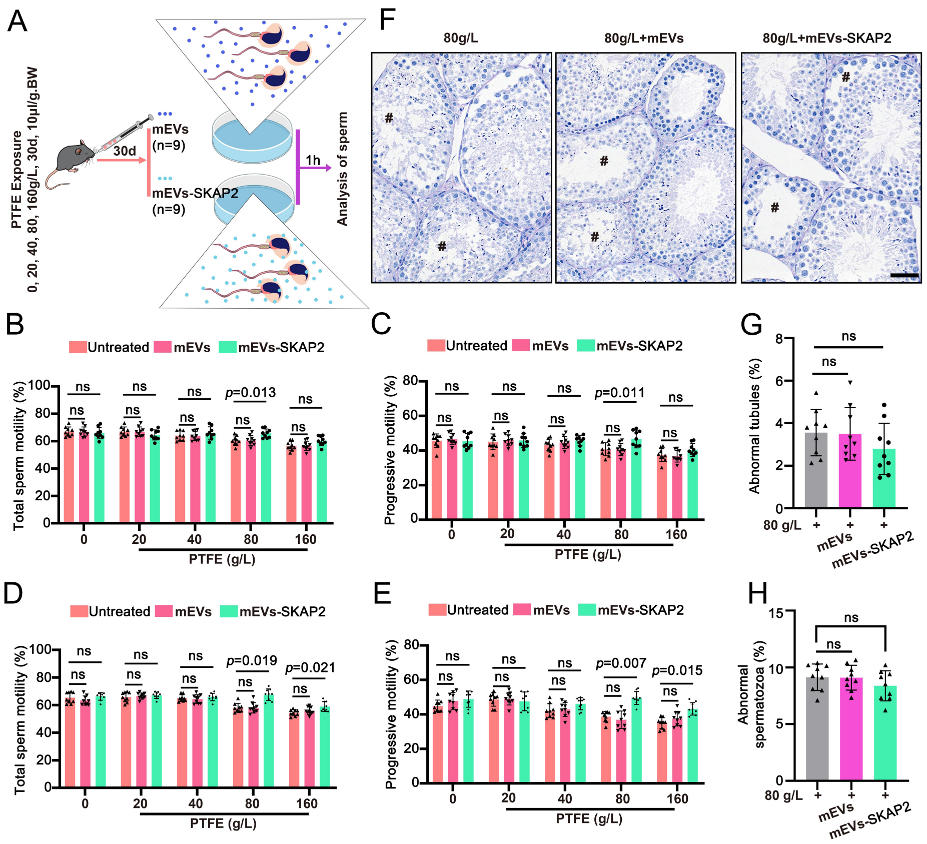

I

The motility level of sperm co-incubation with mEVs or mEVs-SKAP2 in mice exposured to lead.

Characteristics	PR+NP(%)	PR(%)	VAP(μm/s)	VCL(μm/s)	VSL(μm/s)	Hyperactivation(%)
Lead (mean±SD)	22.57±4.55	14.94±2.97	9.80±1.73	21.98±3.18	4.64±1.45	2.79±1.04
Lead+EVs (mean±SD)	22.96±3.13*	15.14±1.97*	10.42±1.84*	23.50±3.91*	4.54±1.06*	1.68±0.73*
Lead+EVs-SKAP2 (mean±SD)	33.91±6.76#	25.92±4.92#	18.22±3.93#	41.24±8.21#	8.68±2.60#	4.08±1.18#
P	<0.001	<0.001	<0.001	<0.001	0.002	0.001

*-Compared with the Lead group, NS: No significance #-Compared with the Lead group, P<0.05

Figure S11. Extracellular vesicles-SKAP2 restores the PTFE and lead-induced decline in mouse sperm quality.

A) A schematic model illustrating the co-incubation of mEVs or mEVs-SKAP2 with mouse sperm. A total of 2.5×10^6 spermatozoa per well were cultured in 1 mL of BWW medium at 37°C with 5% CO_2 for 1 hour in a 24-well plate. B,C) After in vitro co-incubation, CASA analysis of total sperm motility (B) and progressive motility (C) in the untreated group, mEVs, and mEVs-SKAP2 groups. Data are presented as mean ± SD. NS indicates no significant difference. *p*-values were determined using a two-sided Student's *t*-test. D,E) After in vivo injection seven days, CASA analysis of total sperm motility (D) and progressive motility (E) in the untreated group, mEVs, and mEVs-SKAP2 groups. Data are presented as mean ± SD. NS indicates no significant difference. *p*-values were determined using a two-sided Student's *t*-test. F) Periodic acid-Schiff (PAS) staining of untreated group, mEVs, and mEVs-SKAP2 groups mouse seminiferous tubules in vivo efferent ductules injection three days are shown. The # indicate the abnormal tubules. Scale bars = 50 μm. G) Quantification of the ratio of abnormal tubules in untreated group, mEVs, and mEVs-SKAP2 groups after injection through efferent ductules from (F). Data are presented as mean ± SD. *p* values (Student *t*-test, two-sided). H) After in vitro co-incubation 1h, histogram shows the percentage of abnormal spermtozoa in untreated group, mEVs, and mEVs-SKAP2 groups. Data are presented as mean ± SD. *p* values (Student *t*-test, two-sided). I) Statistical analysis of sperm motility parameters after co-incubation of mEVs or mEVs-SKAP2 in mice exposure to lead. *p*-values were determined using a two-sided Student's *t*-test.
